# Transcriptional adaptation of *Mycobacterium tuberculosis* that survives prolonged multi-drug treatment in mice

**DOI:** 10.1128/mbio.02363-23

**Published:** 2023-10-31

**Authors:** Elizabeth A. Wynn, Christian Dide-Agossou, Matthew Reichlen, Karen Rossmassler, Reem Al Mubarak, Justin J. Reid, Samuel T. Tabor, Sarah E. M. Born, Monica R. Ransom, Rebecca M. Davidson, Kendra N. Walton, Jeanne B. Benoit, Amanda Hoppers, Dorothy E. Loy, Allison A. Bauman, Lisa M. Massoudi, Gregory Dolganov, Michael Strong, Payam Nahid, Martin I. Voskuil, Gregory T. Robertson, Camille M. Moore, Nicholas D. Walter

**Affiliations:** 1 Rocky Mountain Regional VA Medical Center, Aurora, Colorado, USA; 2 Department of Biostatistics and Informatics, University of Colorado Anschutz Medical Campus, Aurora, Colorado, USA; 3 Consortium for Applied Microbial Metrics, Aurora, Colorado, USA; 4 Division of Pulmonary Sciences and Critical Care Medicine, University of Colorado Anschutz Medical Campus, Aurora, Colorado, USA; 5 Department of Immunology and Microbiology, University of Colorado Anschutz Medical Campus, Aurora, Colorado, USA; 6 Division of Hematology, Department of Medicine, University of Colorado School of Medicine, Aurora, Colorado, USA; 7 Center for Genes, Environment and Health, National Jewish Health, Denver, Colorado, USA; 8 Department of Medicine, University of Colorado School of Medicine, Aurora, Colorado, USA; 9 Mycobacteria Research Laboratories, Department of Microbiology, Immunology, and Pathology, Colorado State University, Fort Collins, Colorado, USA; 10 Division of Infectious Diseases and Geographic Medicine, Stanford University, Palo Alto, California, USA; 11 Division of Pulmonary and Critical Care Medicine, University of California San Francisco, San Francisco, California, USA; 12 UCSF Center for Tuberculosis, University of California San Francisco, San Francisco, California, USA; Washington University in St. Louis School of Medicine, St. Louis, Missouri, USA

**Keywords:** *Mycobacterium tuberculosis*, gene expression, tolerance, *Mycobacterium*

## Abstract

To address the ongoing global tuberculosis crisis, there is a need for shorter, more effective treatments. A major reason why tuberculosis requires prolonged treatment is that, following a short initial phase of rapid killing, the residual *Mycobacterium tuberculosis* withstands drug killing. Because existing methods lack sensitivity to quantify low-abundance mycobacterial RNA in drug-treated animals, cellular adaptations of drug-exposed bacterial phenotypes *in vivo* remain poorly understood. Here, we used a novel RNA-seq method called SEARCH-TB to elucidate the *Mycobacterium tuberculosis* transcriptome in mice treated for up to 28 days with standard doses of isoniazid, rifampin, pyrazinamide, and ethambutol. We compared murine results with *in vitro* SEARCH-TB results during exposure to the same regimen. Treatment suppressed genes associated with growth, transcription, translation, synthesis of rRNA proteins, and immunogenic secretory peptides. Bacteria that survived prolonged treatment appeared to transition from ATP-maximizing respiration toward lower-efficiency pathways and showed modification and recycling of cell wall components, large-scale regulatory reprogramming, and reconfiguration of efflux pump expression. Although the pre-treatment *in vivo* and *in vitro* transcriptomes differed profoundly*,* genes differentially expressed following treatment *in vivo* and *in vitro* were similar, with differences likely attributable to immunity and drug pharmacokinetics in mice. These results reveal cellular adaptations of *Mycobacterium tuberculosis* that withstand prolonged drug exposure *in vivo,* demonstrating proof of concept that SEARCH-TB is a highly granular pharmacodynamic readout. The surprising finding that differential expression is concordant *in vivo* and *in vitro* suggests that insights from transcriptional analyses *in vitro* may translate to the mouse.

**IMPORTANCE:**

A major reason that curing tuberculosis requires prolonged treatment is that drug exposure changes bacterial phenotypes. The physiologic adaptations of *Mycobacterium tuberculosis* that survive drug exposure *in vivo* have been obscure due to low sensitivity of existing methods in drug-treated animals. Using the novel SEARCH-TB RNA-seq platform, we elucidated *Mycobacterium tuberculosis* phenotypes in mice treated for with the global standard 4-drug regimen and compared them with the effect of the same regimen *in vitro*. This first view of the transcriptome of the minority *Mycobacterium tuberculosis* population that withstands treatment *in vivo* reveals adaptation of a broad range of cellular processes, including a shift in metabolism and cell wall modification. Surprisingly, the change in gene expression induced by treatment *in vivo* and *in vitro* was largely similar. This apparent “portability” from *in vitro* to the mouse provides important new context for *in vitro* transcriptional analyses that may support early preclinical drug evaluation.

## INTRODUCTION

Tuberculosis (TB) is an ongoing health crisis (1). An impediment to TB control is that at least 4–6 months of treatment is needed to reliably cure drug-susceptible TB (2, 3). When treatment starts, *Mycobacterium tuberculosis* (*Mtb*) is highly susceptible to killing. In mice and humans, isoniazid, rifampin, pyrazinamide, and ethambutol (HRZE) eliminate ~99% of culturable bacteria during the initial days- to weeks-long bactericidal phase. Thereafter, the challenge is eradicating the residual sub-population of *Mtb* that withstands treatment despite an absence of drug resistance conferring mutations (4–10). Curing TB more rapidly requires eliminating the small residual *Mtb* population that persists and may lead to treatment failure and relapse (9). Unfortunately, the cellular processes of *Mtb* phenotypes that survive prolonged treatment *in vivo* remain obscure.

Transcriptional profiling has shown how drugs affect bacterial cellular processes *in vitro* (11–17). We are not aware of transcriptional profiling of *Mtb* during drug treatment of laboratory animals, which is important because *Mtb* adapts its cellular processes to physicochemical conditions and immunity encountered in the host (18–26). In the absence of *in vivo* transcriptome data, the degree to which *in vitro* analyses recapitulate changes in gene expression in treated animals has been unclear. Since phenotypic characteristics strongly influence drug susceptibility (27), understanding cellular processes of *Mtb in vivo* would inform drug and regimen development.

**Fig 1:**
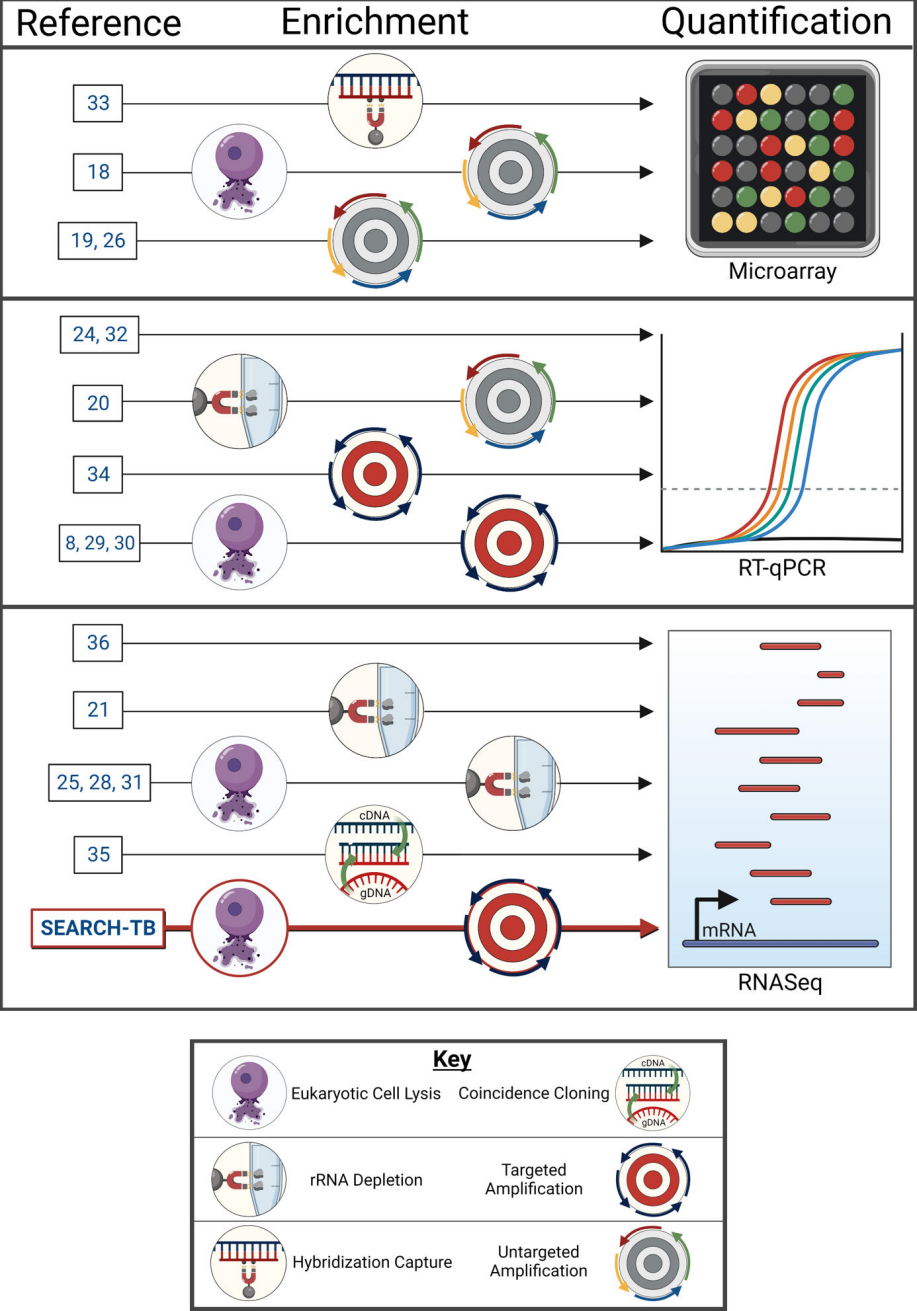
Visual summary of methods previously used to quantify the *Mtb* transcriptome *in vivo*. Each horizontal arrow represents a distinct combination of enrichment and quantification. Varying enrichment methods are represented via the symbols shown in the key. SEARCH-TB is a unique combination of enrichment (eukaryotic cell lysis + targeted amplification) followed by quantification via RNA-seq that has enabled transcriptome evaluation in mice treated for weeks with a potent combination regimen. Image created with Biorender.com.

Transcriptional profiling in animals is impeded by the extremely low abundance of *Mtb* mRNA relative to host RNA. Decreasing bacterial burden during treatment exacerbates the challenge. Diverse methods have been used to first enrich and then quantify *Mtb* mRNA (Fig. 1). Enrichment methods include combinations of differential lysis to separate *Mtb* and eukaryotic cells (8, 18, 25, 28–31), depletion of host and/or bacterial rRNA (21, 25, 28, 31, 32), hybridization capture (33), and selective PCR amplification (8, 29, 30, 34). Quantification methods include microarray (18, 19, 26, 33), multiplex PCR (8, 20, 24, 29, 30, 32, 34), or RNA-sequencing (RNA-seq) (21, 25, 28, 31, 35, 36). Unfortunately, the limited sensitivity of these methods has precluded transcriptional evaluation during long-term drug treatment in animals. Here, we demonstrate SEquening after Amplicon enRiCHment for TB (SEARCH-TB). Using a novel combination of enrichment (differential lysis + targeted amplification) and quantification (RNA-seq), SEARCH-TB achieves sensitivity that will enable routine use of transcriptional profiling for drug evaluation in animal models.

We used SEARCH-TB to evaluate *Mtb* that persists in the lungs of BALB/c mice treated with HRZE for up to 28 days. To establish the baseline state of *Mtb*, we first compared pre-treatment bacterial phenotypes in the mouse versus *Mtb* grown aerobically *in vitro*. We then characterized the transcriptional change induced by HRZE in the mouse and compared it with transcriptional change induced by HRZE *in vitro*.

## RESULTS

### Validation of SEARCH-TB

**Fig 2:**
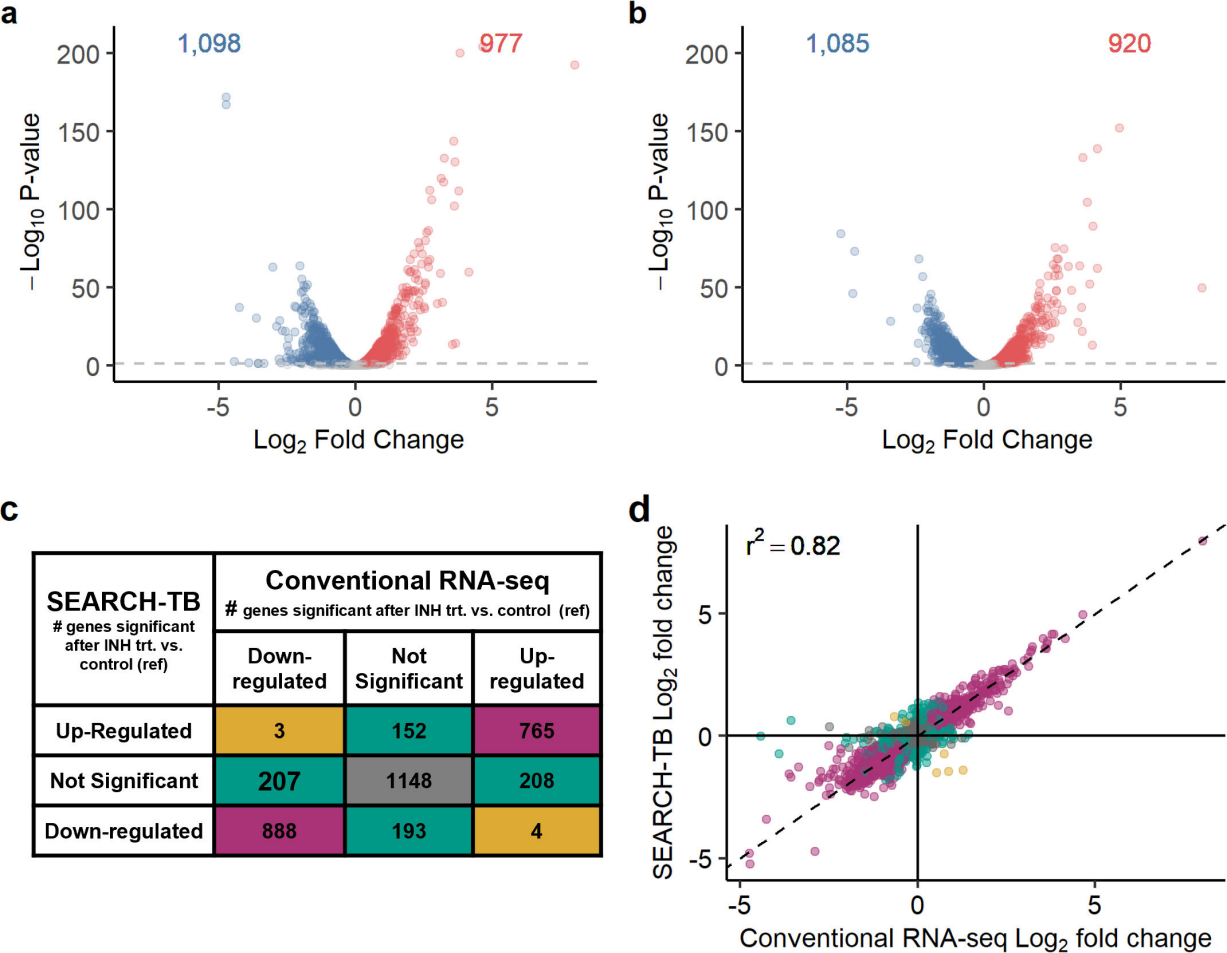
Evaluation of SEARCH-TB platform. (a and b) Volcano plot showing log_2_ fold changes and −log_10_ *P-*values induced by 24 h *in vitro* INH exposure as quantified by RNA-seq (a) and SEARCH-TB (b). Genes significantly down- and up-regulated with INH exposure relative to control (adj. *P* < 0.05) are shown in blue and red, respectively. (c) Comparison of differential expression between INH-treated samples and control samples from RNA-seq or SEARCH-TB data. Purple shading indicates genes with concordant fold-change direction and significance between RNA-seq and SEARCH-TB. Green shading indicates genes that were significant in RNA-seq or SEARCH-TB results but not both. Gold shading indicates genes that were significant for both RNA-seq and SEARCH-TB but in opposite directions. Gray shading indicates genes that were not significantly differentially expressed in either RNA-seq or SEARCH-TB. (d) Comparison of INH versus control fold changes from RNA-seq data versus SEARCH-TB data. Purple, green, gold, and gray colors have the same meaning as in (c).

After exclusions described in Supplemental Information, SEARCH-TB included 3,568 *Mtb* genes. As described in Supplemental Information, we used RNA from 24 h isoniazid exposure *in vitro* to test whether SEARCH-TB provides the same biological information as conventional RNA-seq. The gene sets identified as differentially expressed by SEARCH-TB and conventional RNA-seq strongly overlapped and fold changes showed strong agreement (*R^2^* = 0.82) (Fig. 2), indicating that both platforms provide the same biological information while SEARCH-TB is uniquely capable of profiling in drug-treated animals. Testing also indicated highly repeatable amplification with limited bias (Fig. S3 and S4). The sensitivity of SEARCH-TB relative to existing RNA-seq methods is summarized in Supplemental Information.

### Pre-treatment *Mtb* transcriptome in mice versus *in vitro*

**Fig 3:**
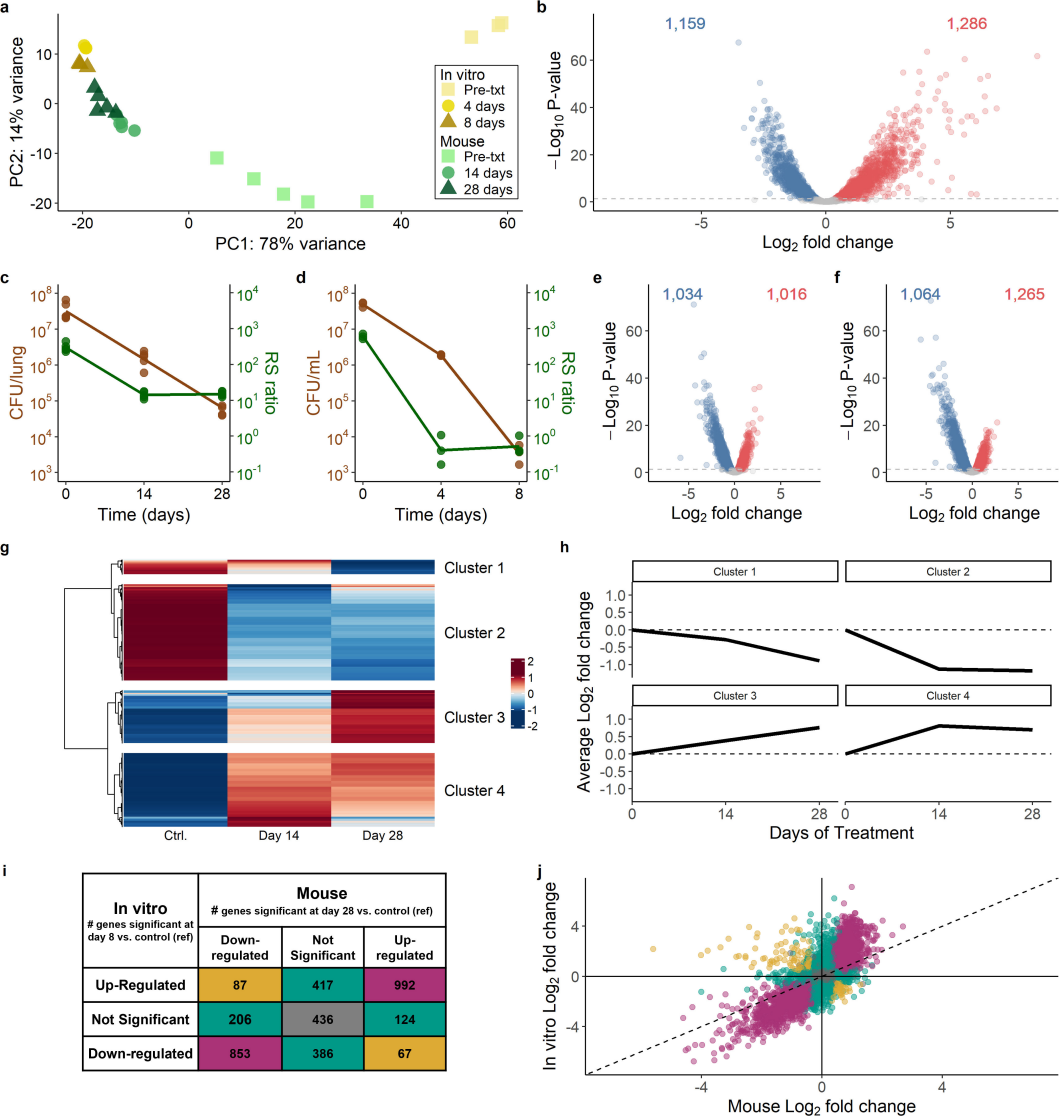
Overview of transcriptional response to HRZE in mice and *in vitro* experiments. (a) The first two principal components of gene expression for mouse and *in vitro* samples. *In vitro* samples are colored using shades of yellow while mouse samples are colored using shades of green. Time points are differentiated by the shade and shape of each point. (b) Volcano plot summarizing the differential expression between *Mtb* in mice and *in vitro* prior to treatment. Genes significantly down- (blue) or up-regulated (red) in mice relative to *in vitro* (adj*. P* < 0.05) are shown. (c and d) *Mtb* CFU and RS ratio values for (c) mouse samples at the pre-treatment control time point and 14 and 28 days after HRZE treatment initiation, and (d) *in vitro* samples at the pre-treatment control time point and 4 and 8 days after HRZE treatment initiation. (e and f) Volcano plots summarizing the differential expression between *Mtb* in (e) 14 days HRZE-treated and control mouse samples and (f) 28 days HRZE-treated and control mouse samples. (g) Estimated gene expression over time in mice for genes significantly differentially expressed between at least two treatment time points (*N* = 2,429). Values are row-scaled, with red and blue indicating higher and lower expression, respectively. Hierarchical clustering of genes identified four broad patterns. (h) Average log_2_ fold changes for each of the four clusters relative to control. Values above and below zero represent up- and down-regulation relative to control, respectively. (i) Comparison of differential expression between mouse (day 28) or *in vitro* (day 8) relative to respective controls. Purple shading indicates genes with concordant fold-change direction and significance between mouse and *in vitro* experiments. Green shading indicates genes that were significant for either mouse or *in vitro* experiments but not both. Gold shading indicates genes that were significant for both mouse and *in vitro* experiments but in opposite directions. Gray shading indicates the genes that were not differentially expressed with HRZE treatment either in mouse or *in vitro* experiments. (j) Comparison of fold changes between mouse (day 28) or *in vitro* (day 8) relative to respective controls. Purple, green, gold, and gray colors have the same meaning as in (i).

We first characterized the baseline pre-treatment state of *Mtb* cellular processes by comparison with *Mtb* grown aerobically *in vitro*. Principal component analysis (PCA) showed that the pre-treatment murine control and the log phase *in vitro* control were distinct (Fig. 3a) with 2,444 (68%) genes differentially expressed (Fig. 3b). Compared with the *in vitro* control*, Mtb* in untreated mice appeared less active with decreased expression of genes associated with rRNA protein synthesis (adj. *P* = 1.7 × 10^−18^), aerobic metabolism (adj. *P* = 2.1 × 10^−5^), fatty acid synthesis (adj. *P* = 0.002), and other growth-associated processes (Online Analysis Tool [https://microbialmetrics.org/analysis-tools/]). Conversely, *Mtb* in untreated mice had higher expression of processes previously associated with adaptation to the host (37), including *dosR* regulon genes that respond to hypoxia, nitric oxide, and carbon monoxide (38, 39) (adj. *P* = 1.4 × 10^−12^); the cholesterol catabolism *kstR1* and *kstR2* regulons (40) (adj*. P* = 7.2 × 10^−5^ and 3.9 × 10^−4^); and mycobactin genes that respond to iron scarcity (41) (adj. *P* = 6.4 × 10^−4^). SEARCH-TB in mice recapitulated previously described *in vivo* persistence adaptations*,* including higher expression of *icl1* (adj. *P* = 3.8 × 10^−13^), the first gene of glyoxylate bypass used during catabolism of fatty acids (42), and increased expression of *tgs1* (adj*. P* = 8.1 × 10^−35^), a gene involved in triacylglycerol synthesis under environmental stress (43).

### Efficacy of HRZE

Treatment efficacy was quantified using traditional and novel molecular pharmacodynamic markers. In mice, 28 days of HRZE treatment reduced CFU 99.8% (7.56 to 4.87 log_10_/lung). *In vitro*, 8 days of HRZE exposure reduced CFU 99.993% (7.7 to 3.57 log_10_/mL). A newer biomarker, the RS ratio (44), declined faster than CFU, indicating that interruption of rRNA synthesis occurs more rapidly than killing, both in mice and *in vitro* (Fig. 3c and d).

### Scale of HRZE-induced expression change in mice and *in vitro*

We began globally by characterizing the scale of expression change induced by HRZE in mice versus *in vitro*. In mice, HRZE significantly altered expression of 2,049 (57%) and 2,329 (65%) genes at days 14 and 28 relative to pre-treatment control, respectively (Fig. 3e and f). Hierarchical clustering identified genes with similar expression changes over time (Fig. 3g). Most transcriptional changes occurred by day 14 (Fig. 3h, Clusters 2 and 4). A smaller gene subset changed more gradually (Fig. 3h, Clusters 1 and 3). *In vitro* HRZE exposure significantly altered expression of 2,748 (77%) and 2,800 (78%) genes at days 4 and 8 relative to control, respectively.

### Comparison of transcriptional changes induced by HRZE in mice and *in vitro*

There were broad similarities and limited but important differences between the effects of HRZE in mice and *in vitro*. At the latest treatment time points (8 days *in vitro* and 28 days in mice), differential expression was largely concordant. Overall, 64% of genes had the same significance results *in vitro* and in the mouse, relative to pre-treatment (Fig. 3i). Only 4% of genes were significantly differentially expressed in opposite directions (colored gold in Fig. 3i and j), indicating differing effects of HRZE in mice and *in vitro*. The specific processes that differ between mice and *in vitro* are highlighted throughout the following sections. Notably, fold changes were more extreme *in vitro* than in mice (Fig. 3j).

### Effect of HRZE on the *Mtb* cellular processes

**Fig 4:**
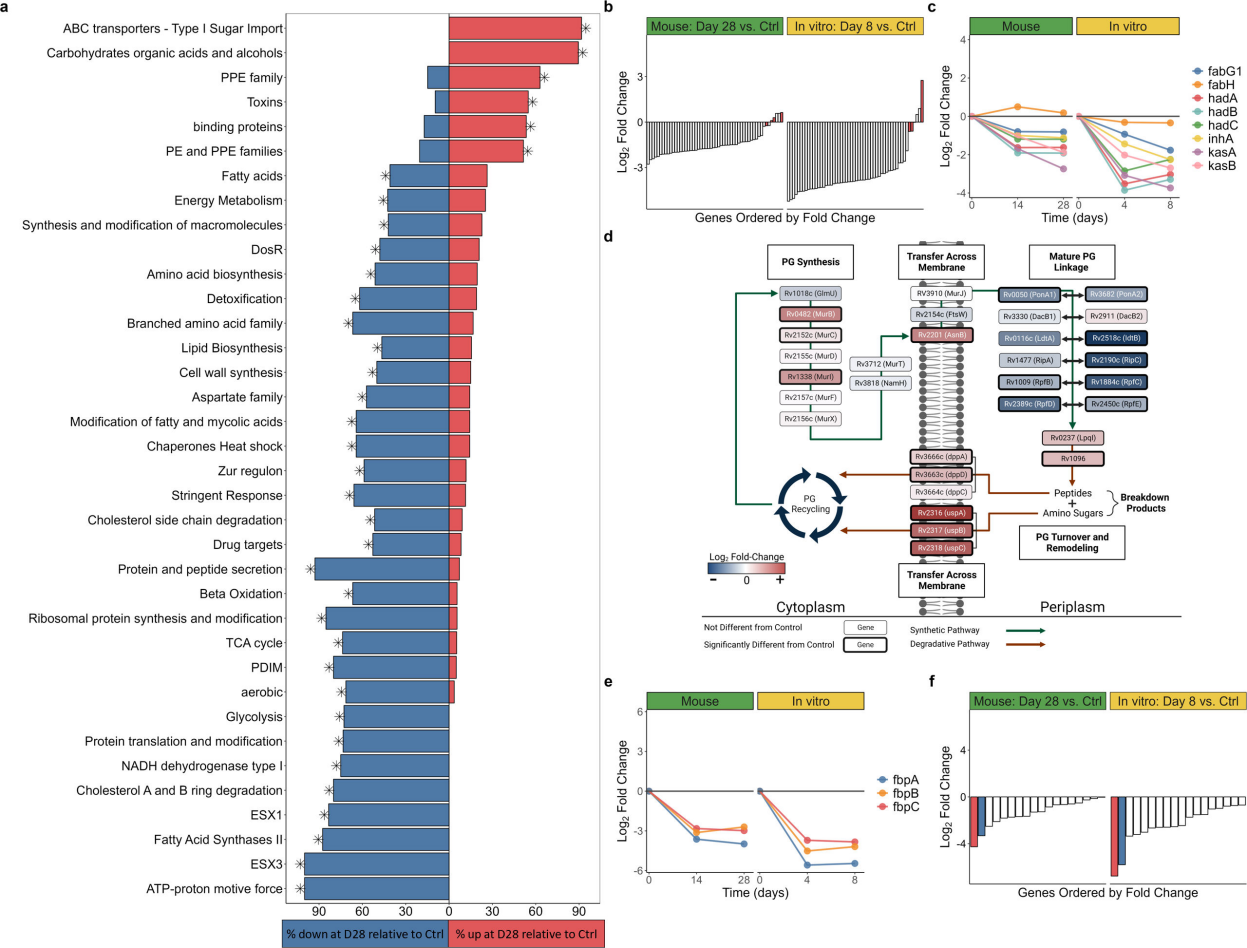
Summary of gene set enrichment and transcriptional changes in biological processes. (a) Gene categories significantly enriched for genes differentially expressed between day 28 and control murine samples. The percentage of genes in each category significantly up- (red) or down-regulated (blue) for each comparison is illustrated. Asterisks indicate statistical significance (adj*. P* < 0.05). (b) Fold change of ribosomal protein genes in mice on day 28 (left) and *in vitro* (right) on day 8, relative to control. Red bars indicate the four alternative C-ribosomal protein paralogs. (c and e) Fold-change values in mice at days 14 and 28 (left) and *in vitro* (right) at days 4 and 8, relative to control for (c) FAS-II and (e) antigen 85 gene sets. (d) Graphical representation of changes in the peptidoglycan synthesis, modification, and recycling pathways in mice on day 28 relative to control. Log_2_ fold-change values for genes in the process are indicated by the color of each box and the thick outlines of boxes represent genes that are significantly differentially expressed. Figure adapted from Maitra et al., 2019 (45). Image created with Biorender.com. (f) Fold change of ESX-1 genes in mice on day 28 (left) and *in vitro* (right) on day 8, relative to control. Red bar represents *esxA* and blue bar represents *esxB*.

Since our focus is *Mtb* phenotypes that withstand prolonged drug exposure, we describe expression changes at the latest time point (28 days in mice or 8 days *in vitro*) relative to control, unless otherwise noted. Other time points can be explored via the Online Analysis Tool (https://microbialmetrics.org/analysis-tools/). After 28 days of HRZE treatment in mice, 36 of 124 functional categories (Table S3) were significantly enriched (Fig. 4a).

### Transition to a quiescent bacterial phenotype

#### Decreased growth and macromolecule synthesis

HRZE decreased expression of genes associated with growth and macromolecule synthesis. Specifically, treatment suppressed ribosomal protein gene expression in mice (adj. *P* = 2.9 × 10^−15^) and *in vitro* (adj*. P* = 1.0 × 10^−14^) relative to control (Fig. 4b), consistent with decreased ribosome synthesis, a process fundamentally coupled with bacterial replication (46, 47). An exception was the four alternative C-ribosomal protein “remodeling” paralogs lacking the zinc-binding CXXC motif (*rpmB1*, *rpsR2*, *rpsN2*, and *rpmG1*), which had sustained or significantly increased expression relative to control (Fig. 4b), consistent with ribosomal remodeling by less-active *Mtb*.

Slowing of protein synthesis was suggested by decreased expression of the protein translation and modification category that includes genes responsible for translational initiation, promotion of tRNA binding, elongation, termination, and protein folding (adj *P* = 0.006 in mice; adj. *P* = 0.007 *in vitro*). Transcriptional slowing was suggested by decreased expression of *gyrA* and *gyrB* in mice and *in vitro* (least significant adj*. P* = 1.8 × 10^−5^).

#### Decreased cell wall synthesis but continued remodeling and recycling

To summarize the effect of HRZE on cell wall biosynthesis, we evaluated expression change for the major cell wall constituents: mycolic acids, phthiocerol dimycocerosates (PDIM), peptidoglycan, and trehalose.

##### Mycolic acids

Slowing of the first step of mycolic acid synthesis was indicated by the decreased expression of Rv2524c (*fas),* the gene coding for fatty acid synthetase I, in mice (adj. *P* = 1.7 × 10^−9^) and *in vitro* (adj*. P* = 3.2 × 10^−9^) and the decreased expression of the *fas* transcriptional regulator Rv3208 (48) in mice (adj*. P* = 1.9 × 10^−4^) and *in vitro* (adj*. P* = 1.1 × 10^−11^). Genes coding for the second step of elongation of acyl-coenzyme A to long-chain fatty acids by fatty acid synthetase II were also decreased in both mice (adj*. P* = 0.011) and *in vitro* (adj*. P* = 0.027) (Fig. 4c). Finally, genes associated with elongation, desaturation, modification, and transport of the mature mycolic acids to the cell wall had decreased expression both in mice and *in vitro* (Online Analysis Tool).

##### PDIM

Genes involved in synthesis of PDIM, the outer surface glycolipids important for intracellular survival and virulence, were down-regulated in mice (adj*. P* = 9.9 × 10^−5^) and *in vitro* (adj*. P* = 0.017).

##### Peptidoglycan

In contrast to mycolic acids and PDIM, certain genes involved in peptidoglycan synthesis, modification, and recycling had increased expression. Specifically, in HRZE-treated mice, all five peptidoglycan synthesis genes assayed in the division cell wall operon (*ftsQ*, *murC*, *ftsW*, *murD*, *murR*, and *murE*) had significantly increased expression at day 14 and three remained significantly up-regulated at day 28 (Online Analysis Tool). Active recycling was further suggested by significantly increased expression in mice and *in vitro* of genes coding for the UspABC amino-sugar importer and the DppABCD dipeptide importer that transport peptidoglycan breakdown products (Fig. 4d) (49).

##### Trehalose

The *treY/Z* genes that synthesize the essential metabolite and cell wall constituent trehalose from glycogen were significantly up-regulated at all post-treatment time points in mice and *in vitro*. Two other trehalose synthesis pathways (*otsA/B* and *glgE/treS*) were not differentially expressed in mice or *in vitro*. Remodeling of the trehalose component of the cell envelope was suggested by increased expression of Rv3451 (*cut3*) in mice (adj*. P* = 0.002) and *in vitro* (adj*. P* = 4.2 × 10^−4^), which codes for a stress-responsive trehalose dimycolate hydrolase (50). Additionally, HRZE induced significantly increased expression in mice and *in vitro* of four of the five genes coding for the LpqY/SugABC importer that is specific for the transport of trehalose (51).

### Decreased expression of genes for secretory peptides and immunogenic proteins

Genes for the antigen 85 complex, a secreted protein essential for survival within macrophages (52), were suppressed by HRZE in mice (least significant adj*. P* = 3.1 × 10^−24^) and *in vitro* (least significant adj*. P* = 2.6 × 10^−31^) (Fig. 4e). Among the type VII secretion systems that modulate immunity, the ESX-1 locus was down-regulated (adj*. P* = 9.4 × 10^−5^ in mice; adj*. P* = 1.3 × 10^−5^ *in vitro*) with particularly strong suppression of *esxA* and *esxB,* genes coding for highly immunogenic early secretory antigenic 6 kDa (ESAT-6) and culture filtrate protein 10 (CFP-10), in mice (least significant adj*. P* = 4.7 × 10^−24^) and *in vitro* (least significant adj*. P* = 2.4 × 10^−35^) (Fig. 4f). All ESX-3 locus genes assayed were significantly down-regulated with particularly strong suppression of immunogenic *esxG* and *esxH* in mice (least significant adj*. P* = 1.1 × 10^−34^) and *in vitro* (least significant adj*. P* = 4.0 × 10^−32^). In contrast, at least six of the seven genes in the ESX-4 locus, recently described as having “wholly unknown” function (53), had significantly increased expression in mice and *in vitro*.

### Active bacterial adaptations to drug stress

#### Metabolic adaptation

##### Electron transport and aerobic respiration

**Fig 5:**
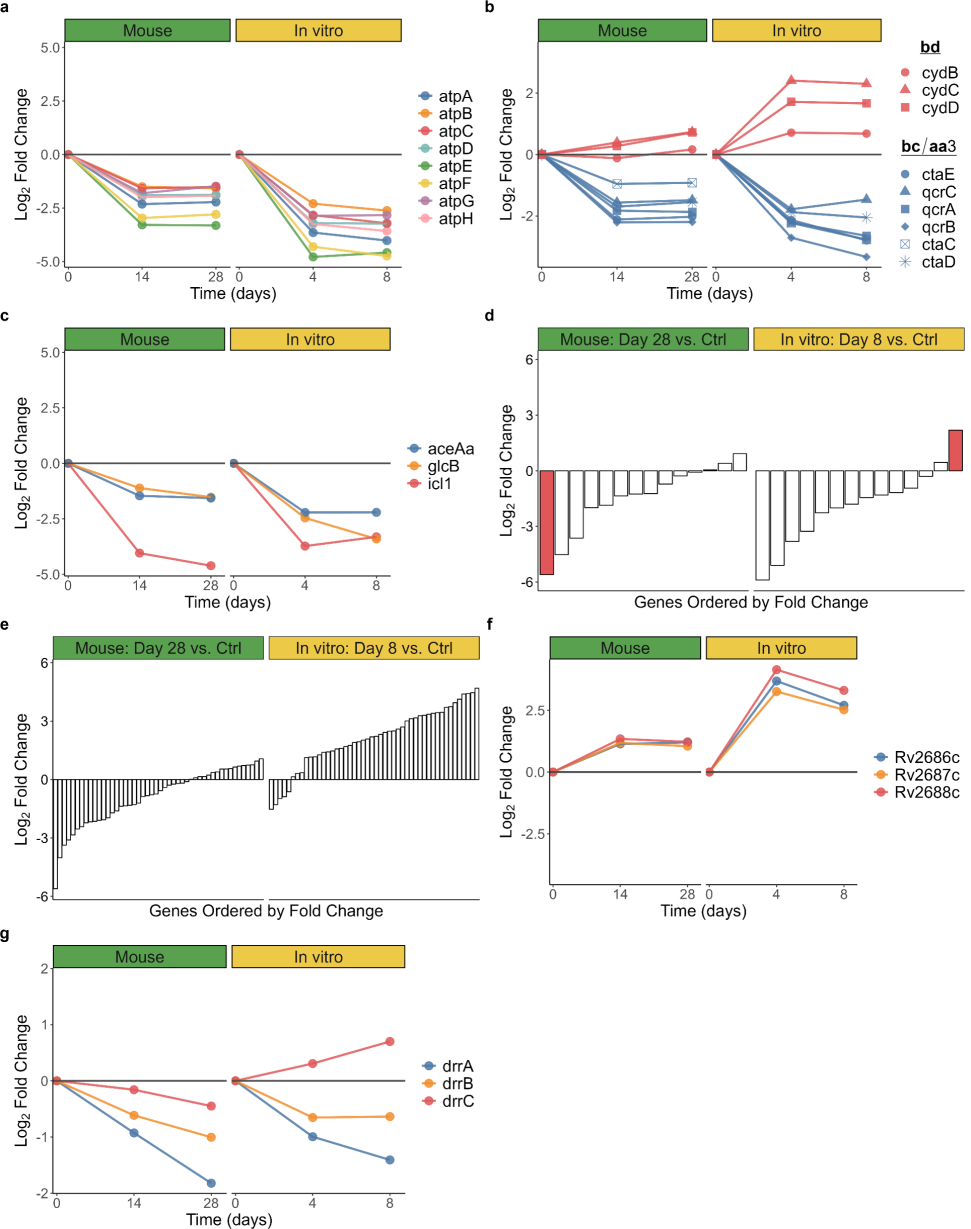
Summary of transcriptional changes in biological processes. Fold change in mice (left) and *in vitro* (right), relative to control for (a) ATP synthetase, (b) cytochrome bcc/aa3 supercomplex and the bd oxidase, (c) glyoxylate bypass, (d) heat shock proteins (red bar represents *hspX*), (e) dosR, (f) the Rv2686c-2688c efflux pump, and (g) DrrABC efflux pump gene sets.

HRZE suppressed expression of all genes coding for ATP synthetase in mice (adj*. P* = 7.6 × 10^−4^) and *in vitro* (adj*. P* = 0.003) (Fig. 5a). Oxidative phosphorylation appeared to transition from the primary cytochrome *bcc/aa3* supercomplex (down-regulated) to the less-efficient cytochrome *bd* oxidase (up-regulated) that has been implicated in persistence under environmental and drug stress (54) (Fig. 5b). TCA cycle genes were down-regulated with HRZE treatment in mice (adj*. P* = 0.001) and *in vitro* (adj*. P* = 0.010). Genes coding for NADH dehydrogenase types I and II and succinate dehydrogenase types I and II were also down-regulated (Online Analysis Tool). By contrast, three of the four fumarate reductase genes were significantly induced by HRZE in mice, and all four were significantly induced by HRZE *in vitro* (Online Analysis Tool).

##### Central carbon metabolism

Respiratory slowing was not accompanied by increased expression of glyoxylate bypass genes, an alternative pathway previously implicated in drug tolerance (42). Instead, HRZE treatment suppressed the gene for isocitrate lyase (*icl1*), the first step of glyoxylate bypass, both in mice (adj*. P* = 1.9 × 10^−37^) and *in vitro* (adj*. P* = 2.0 × 10^−14^) (Fig. 5c). The gene coding for the alternative isocitrate lyase (*aceAa*) was also significantly down-regulated in mice (adj*. P* = 7.3 × 10^−13^) and *in vitro* (adj*. P* = 3 × 10^−15^) after treatment with HRZE. Genes associated with carbon storage such as triacylglycerol had discordant regulation in mice and *in vitro*. Specifically, *tgs1,* a gene in the DosR regulon which codes for triacylglycerol synthase, decreased 4.4-fold with HRZE treatment (adj. *P* = 7.0 × 10^−13^) in mice but increased 10.9-fold (adj. *P* = 3.2 × 10^−16^) *in vitro*.

### Discordant expression of DosR regulon and other stress responses in mice and *in vitro*

Although heat shock proteins (HSPs) that act as chaperones in protein folding are often described as a stress response, HRZE suppressed expression of HSP genes. A notable discordance between murine and *in vitro* results is the hypoxia-responsive *hspX* (noted in red in Fig. 5d) that had the greatest negative fold change of all genes evaluated in mice (48.8-fold decrease, adj*. P* = 4.8 × 10^−57^) yet was significantly up-regulated with HRZE treatment *in vitro* (4.6-fold increase, adj. *P* = 2.0 × 10^−8^). Concordant with this result, HRZE repressed DosR expression in mice (adj*. P* = 0.032) but induced the DosR regulon *in vitro* (adj*. P* = 2.2 × 10^−7^) (Fig. 5e). HRZE was associated with down-regulation of genes of the stringent response, typically induced by nutrient deprivation and other environmental stresses, in mice (adj*. P* = 2.9 × 10^−15^) and *in vitro* (adj*. P* = 1.6 × 10^−14^). Similarly, HRZE decreased expression of *relA* which codes for the stringent response regulator, in mice (adj*. P* = 2.0 × 10^−6^) and *in vitro* (adj*. P* = 7.0 × 10^−6^).

### Transcriptional and post-transcriptional reprogramming

SEARCH-TB indicated large-scale regulatory reprogramming. For example, HRZE changed most of the 188 transcription factors assayed in mice, with 66 significantly increased and 47 significantly decreased. The regulatory perturbation was even more pronounced *in vitro* with 87 significantly increased and 55 significantly decreased transcription factors. Of the 12 sigma factor genes included in SEARCH-TB, *sigF, sigI*, and *sigM* were significantly up-regulated following HRZE treatment in both mice and *in vitro* while *sigA, sigB, sigD,* and *sigK* were significantly down-regulated with HRZE treatment in both mice and *in vitro*. HRZE significantly altered expression of *sigC* and *sigL* in both mice and *in vitro* but in discordant directions, suggesting differing regulatory responses *in vivo* and *in vitro*.

HRZE appeared to activate the post-transcriptional toxin–antitoxin system that modulates the concentration of existing transcripts. Toxin genes were induced in mice (adj*. P* = 0.011) and *in vitro* (adj*. P* = 0.027). The counter-regulatory antitoxins that restrict toxin activity were not categorically altered in mice but were significantly suppressed *in vitro* (adj*. P* = 0.037).

### Adaptation of efflux pump expression

HRZE altered expression of many efflux pumps, in varying directions (Table S4). As examples, Rv2686c-2688c, which is associated with fluoroquinolone tolerance (55), was up-regulated with HRZE treatment in mice and *in vitro* (Fig. 5f), but the genes coding for DrrABC, which is associated with daunorubicin tolerance and appears in some clinical drug-resistant strains (55), were down-regulated (Fig. 5g).

### Expression of drug targets

Because processes up-regulated during drug exposure may represent survival mechanisms that could be targeted to eradicate persisting *Mtb,* we evaluated genes coding for the known targets of 31 established and investigational new drugs (36 genes) (Table S5). These genes were predominantly suppressed in mice (19 down-regulated and three up-regulated) and *in vitro* (26 down-regulated and two up-regulated). Exceptions to the prevailing down-regulation were increased expression of the genes coding for the Mur ligases B and C that initiate peptidoglycan synthesis in mice (56) and *rfe,* the gene coding for the phosphoglycosyltransferase WecA that initiates arabinogalactan synthesis with HRZE treatment in mice and *in vitro* (57).

### Differences between *Mtb* in mice and *in vitro* at final time points

After 8 days *in vitro* and 28 days in the mouse, *Mtb* transcriptomes were more similar than they were prior to HRZE exposure (Fig. 3a). Nonetheless, 1,014 genes (28%) remained differentially expressed between the final murine and *in vitro* time points evaluated in this study.

## DISCUSSION

SEARCH-TB elucidated cellular adaptations of *Mtb* that withstand long-term HRZE treatment. After 28 days of treatment—which reduced culturable *Mtb* in mouse lungs by 99.8%—SEARCH-TB indicated broad suppression of cellular activity including slowing of metabolism, reduced synthesis of macromolecules, and reduced secretion of immune-modulating peptides. SEARCH-TB also suggested bacterial adaptation to drug stress, including a shift in electron transport to the alternative, less efficient cytochrome *bd* oxidase, ribosomal remodeling, cell wall remodeling and recycling, and reprogramming of regulatory and efflux pump activity. Importantly, the effects of HRZE in mice and *in vitro* were broadly similar with some differences that likely reflect effects of pharmacokinetics (PK), immunity, and *in vivo* environment. By quantifying extremely low-abundance *Mtb* transcripts in mice, SEARCH-TB should enable a new era of molecular evaluation of drug effect *in vivo*.

Differences between mice and *in vitro* before treatment reflect bacterial adaptation to immunity and the lung environment, consistent with a recent review (37) of the treatment-naïve *in vivo Mtb* transcriptome, both in terms of broad cellular processes (down-regulation of genes associated with transcription, translation, and metabolism) and specific adaptations (e.g., increased expression of DosR genes, glyoxylate bypass genes). Importantly, the untreated mouse was the starting point for this study. As highlighted below, drug exposure elicited transcriptional adaptations that diverge from well-established transcriptional adaptations to environmental stress (such as pH, hypoxia, and nutrient starvation).

SEARCH-TB indicated that HRZE suppressed *Mtb* growth and metabolism, consistent with previous *in vitro* analyses of drug effects (11–17). There was a general decrease in the expression of genes involved in the synthesis of all major macromolecules. The transition to a less-active bacterial phenotype observed in mice treated with HRZE is similar to the expression change observed in sputa of humans treated with HRZE (8, 18). There was also decreased protein and peptide secretion, including Ag85 and the ESX1 Type VII secretion system that exports the immunogenic ESAT-6 and CFP-10 proteins, suggesting that drug stress might alter the pathogen’s capacity to adapt to host immunity. Consistent with the current mouse data, our previous study of human sputum showed that treatment with HRZE significantly decreased expression of Ag85 genes and the genes (*esxA* and *esxB*) coding for ESAT-6 and CFP-10 (8).

Nonetheless, SEARCH-TB suggested that drug-stressed *Mtb* is not inert or incapacitated. Increased expression of genes associated with peptidoglycan and trehalose synthesis and recycling suggest ongoing cell wall modification. SEARCH-TB indicated transcriptional and post-transcriptional regulatory reprogramming and altered efflux pump expression. Metabolism and energy generation appeared reconfigured away from high respiratory activity that maximizes ATP generation, toward reduced respiratory efficiency and oxidative phosphorylation activity.

Prior to treatment with HRZE, DosR regulon genes that respond to impaired respiration (58) were expressed on average >10-fold more highly in mouse lung than under aerobic *in vitro* conditions*,* consistent with bacterial adaptation to host-derived nitric oxide and hypoxia by intracellular *Mtb* in the mouse. Strikingly, during treatment with HRZE, expression of the DosR regulon decreased significantly in mice and increased significantly *in vitro*. The decrease in DosR expression in mice is consistent with our previous observation that HRZE suppressed DosR expression in human sputum (8). We hypothesize that, during the first month of treatment, host immune response to the pathogen is modulated by the >99% reduction in *Mtb* burden and decreased bacterial secretion of immunogenic peptides. A less-intense inflammatory response with diminished macrophage and neutrophil activation would decrease nitric oxide exposure, reducing the need for *Mtb* DosR expression. This hypothesis that DosR may serve as an indirect readout of immunity is consistent with our previous observation that the DosR regulon has lower expression in TB patients with AIDS than in immunocompetent patients (29) and with our finding that *in vitro*, in the absence of immunity, HRZE increased rather than decreased expression of genes in the DosR regulon.

Also notable was decreased expression of the gene coding for isocitrate lyase, the first step of the glyoxylate bypass which is required to establish productive infection in animals (59–61). Consistent with prior results (59–61), *icl1* was expressed more highly in untreated mice than *in vitro. icl1* was shown to increase with sublethal exposure to rifampin, INH, or streptomycin *in vitro* (42). By contrast, we found that lethal doses of HRZE strongly suppressed, rather than induced, expression of *icl1* and *aceAa* which codes for an alternative isocitrate lyase. This is consistent with our previous observation that expression of *icl1* and *aceAa* declined significantly in human sputum during treatment with HRZE (8). This highlights that adaptations to HRZE differ from adaptations to host environments that enable persistent infection in the absence of drug therapy and that the response to exposure to a combination regimen *in vivo* may differ from single drug exposures *in vivo*.

Drugs have historically targeted growth-associated cellular processes (62) that SEARCH-TB showed to be down-regulated after HRZE treatment. While transcriptional down-regulation of drug targets does not necessarily mean that a drug will be ineffective, genes such as those coding for Mur ligases and WecA that showed increased expression after 1 month of HRZE *in vitro* could indicate adaptations that enable *Mtb* to withstand drug exposure. Similarly, genes coding for the alternative cytochrome *bd* oxidase that is a proposed drug target (63) were up-regulated at a time when metabolism was globally suppressed.

A finding with important implications is the concordance of murine and *in vitro* results. Although *Mtb* cellular processes were very different in mice and *in vitro* before treatment, HRZE induced broadly similar changes *in vivo* and *in vitro*. This indicates that drug exposure is a stress of sufficient intensity that it overwhelms phenotypic differences that exist due to differences in environmental conditions present *in vitro* or in a mouse. One exception to the similarity is that the magnitude of fold change was smaller in mice than *in vitro*. This likely reflects diminished drug/target engagement (i.e., decreased drug exposure) in a mouse versus *in vitro* due to *in vivo* PK. A second exception was the discordance in the effect of HRZE on DosR expression (down in mice, up *in vitro*) as discussed above. Nonetheless, our observation that differential expression is largely consistent despite baseline differences in environment and phenotype provides new context for interpretation of transcriptome data from *in vitro* drug exposure experiments.

As a readout of the effect of drugs on *Mtb* cellular processes, SEARCH-TB could improve precision of pharmacodynamic evaluation, providing greater information than culture-based enumeration of bacterial burden (the existing standard method) (64). We have previously shown that CFU does not capture the entirety of complex drug effects *in vivo*. For example, TB regimens that have identical effects on CFU in mice can have different long-term relapse outcomes (44, 65). Our previous RS ratio studies provided proof of concept that molecular measures of bacterial cellular processes *in vivo* can distinguish regimens that are indistinguishable based on CFU (44, 65). SEARCH-TB advances molecular characterization of drug effects *in vivo* to a higher level of granularity. Others have demonstrated the power of *Mtb* transcriptional readouts of drug effect to predict drug interactions *in vitro* (66). SEARCH-TB may enable assessment of drug interactions and regimens based on molecular effects on cellular processes *in vivo*.

This report has several limitations. First, specific and efficient primers could not be designed for ~12% of *Mtb* transcripts. Second, as discussed in Supplemental Information, between-target variation in the amplification efficiency of SEARCH-TB primers likely affects the rank-order of gene counts, meaning that, for individual genes, a higher absolute count does not necessarily indicate greater expression. However, because amplification is highly repeatable, the modest amplification bias identified does not affect estimation of differential expression between groups. Indeed, we showed that SEARCH-TB provides the same biological interpretation as conventional RNA-seq. Third, transcriptional profiling is inherently unable to resolve the enduring question of whether the sub-population that persists late into treatment results from selection (i.e., elimination of easily killed *Mtb*) or physiological adaptation of extant *Mtb*. Fourth, there are important differences between *in vitro* and mouse conditions that we could not control. For example, *in vitro* exposure conditions employed herein do not recapitulate the dynamic PK drug profiles that occur in mice. It is also noted that pyrazinamide is active in the mouse but is not expected to be active *in vitro* under the conditions tested herein. The observation that differential expression is similar *in vitro* and in the mouse despite these differences indicates that the effect of HRZE on the transcriptome is largely independent of the starting bacterial phenotype. Finally, here we evaluated one regimen in one murine model. Next steps include evaluation of individual drugs and diverse regimens in additional animal models.

SEARCH-TB elucidated the effect of prolonged drug treatment on *Mtb* transcription in animal models*,* revealing adaptations distinct from those observed under environmental stress. *Mtb* that survived 1 month of HRZE treatment appeared substantially less active than prior to treatment but was not inert, with transcriptional changes suggesting adaptation for survival. SEARCH-TB should enable more informative *in vivo* molecular pharmacodynamic evaluation that accelerates identification of new highly potent regimens.

## MATERIALS AND METHODS

### Design of the SEARCH-TB assay

The SEARCH-TB assay uses an AmpliSeq for Illumina custom pool designed to amplify coding sequences (CDS) of *Mtb* complex (MTBC) organisms. To assure amplification across diverse lineages, the design used eight MTBC reference genomes (Table S1). Annotations were prepared for *Mtb* H37Rv (NC_000962) and *Mtb* Erdman (AP012340.1) as described in the Supplemental Information. To avoid off-target amplification (i.e., of nonMTBC organisms), primers were cross-referenced during design with 12 “exclusion” genomes that included phylogenetically diverse bacteria as well as human and mouse (Table S2).

### Evaluation of amplification bias

We tested for amplification bias (i.e., differences in amplification efficiency between primer pairs targeting different *Mtb* sequences) using replicate human lung RNA samples spiked with *Mtb* genomic DNA (gDNA) (Supplemental Information). Since all targeted sequences are present as single copies in gDNA, an entirely unbiased assay would hypothetically result in the same copy number for all targets, indicating that all primer pairs amplified with identical efficiency. We defined amplification bias as deviation from this ideal by comparing the observed expression for a gene to the expected expression assuming no amplification bias (Supplemental Information).

### Evaluation of repeatability of amplification

To evaluate repeatability, we spiked 1 pg *Mtb* RNA into 1 ng human lung RNA (Supplemental Information). We compared counts per million values for each gene between technical replicates. We also evaluated repeatability over time by prepping and sequencing 19 of the *in vitro* and murine samples described below two times with up to a 4-month intervening interval. We quantified batch effect by calculating the difference in expression (normalized with DESeq2’s variance stabilizing transformation) (67) of each gene between replicate pairs, then averaging across replicates. We compared the magnitude of the batch effect with the observed treatment effect.

### Concordance of SEARCH-TB with conventional RNA-seq

We evaluated whether SEARCH-TB identified the same transcriptional changes as a conventional RNA-seq method without *Mtb*-targeted amplification (Illumina TrueSeq) after 24 h *in vitro* isoniazid (INH) exposure (Supplemental Information). We first evaluated RNA from control (*N* = 4) and INH-treated samples (*N* = 4) via conventional RNA-seq to serve as a reference standard. We then spiked the same RNA from control and INH-treated *Mtb* into human lung RNA at a ratio of 1:1,000 and sequenced it via SEARCH-TB. After calculating differential expression between control and INH-treated samples separately for conventional RNA-seq and SEARCH-TB using edgeR (68), we compared the significant genes and fold changes identified by the two platforms.

### *In vitro* experiments

*Mtb* strains H37Rv and Erdman were cultured *in vitro* using Middlebrook 7H9 broth (Difco) supplemented with 0.085 g/L NaCl, 0.2% glucose, 0.2% glycerol, 0.5% BSA, and 0.05% Tween-80. All culturing was performed at 36.5°C and 5.0% CO_2_. Single-use frozen *Mtb* aliquots were revived in 7H9 and grown to mid-log phase, then cultures were diluted to OD_600_ = 0.05, dispensed in 5.0 mL aliquots into sterile glass tubes (20 × 125 mm) containing sterile stir bars (12 × 4.5 mm), and outgrown for 18 h under rapid agitation (~200 rpm stirring speed using a rotary magnetic tumble stirrer) prior to the initiation of drug exposure. RNA was collected from *Mtb* H37Rv exposed to INH or *Mtb* Erdman exposed to HRZE *in vitro* (Supplemental Information).

### Murine drug experiments

All animal procedures were conducted according to relevant national and international guidelines and approved by the Colorado State University Animal Care and Use Committee as described in the Supplemental Information. Briefly, female BALB/c mice, 6 to 8 wk old, were exposed to aerosol (Glas-Col) with *Mtb* Erdman strain resulting in the deposition of 4.55 ± 0.03 (SEM) log_10_ CFU in lungs 1 day following aerosol. After 11 days, five mice were euthanized to serve as the pre-treatment control group. Groups of five mice each were treated with HRZE at standard doses 5 days a week for 14 or 28 days before euthanasia. Lungs were aseptically dissected and flash frozen in liquid nitrogen before processing.

### RNA extraction, sequencing, and data preparation

RNA extraction, library preparation, sequencing, and data preparation are detailed in the Supplemental Information.

### Statistical analysis of murine and *in vitro* experiments

Murine and *in vitro* sequence data were analyzed together using edgeR (68, 69) to identify the effect of HRZE treatment and compare gene expression between murine and *in vitro* experiments. We fit negative binomial generalized linear models to each gene and included terms for murine and *in vitro* time points (control, day 14 and day 28 in mice; control, day 4 and day 8 *in vitro*). Likelihood ratio tests were performed to compare expression between murine time points, between *in vitro* time points, and between murine and *in vitro* experiments before and at the end of treatment. Genes with Benjamini–Hochberg adjusted *P*-value (70) less than 0.05 were considered significant. For murine experiments, we used hierarchical clustering to identify groups of genes with similar changes in gene expression over the course of treatment as follows. First, for genes that were differentially expressed between at least two-time points, we calculated the expected expression at each time point using the edgeR models. Then, the expected expression values were hierarchically clustered based on Euclidian distance using Ward’s method (71) to find clusters of genes with similar patterns of expression over time.

PCA was performed using data for the 500 most variable genes after normalization with DESeq2’s variance stabilizing transformation (67).

Using hypergeometric tests in the hypeR R package (72), we performed functional enrichment for each pairwise combination of murine and *in vitro* time points to evaluate whether differentially expressed genes were overrepresented in gene categories established by Cole et al. (73) or curated from the literature (Table S3). Enrichment analysis was run twice for each pairwise combination, first using significantly up-regulated genes and then using significantly down-regulated genes. Gene categories with <8 genes were excluded. Gene categories with Benjamini–Hochberg adjusted *P*-values (70) less than 0.05 were considered significant. All analyses used R (v4.1.1) (74).

### Online analysis tool

Differential expression, functional enrichment, and visualizations can be evaluated interactively using an Online Analysis Tool (https://microbialmetrics.org/analysis-tools/) created using the R package Shiny (75).

## Data Availability

All raw sequencing data have been deposited in the Sequence Read Archive (SRA) under BioProject accession PRJNA939248. Individual samples have BioSample accession numbers SAMN33461189 through SAMN33461251.

## SUPPLEMENTAL MATERIAL

Supplemental Information mbio.02363-23-s0001.docx Additional information on methods and results.

ASM does not own the copyrights to Supplemental Material that may be linked to, or accessed through, an article. The authors have granted ASM a non-exclusive, world-wide license to publish the Supplemental Material files. Please contact the corresponding author directly for reuse.
