# Supplementary material for: Transcriptional adaptation of Mycobacterium tuberculosis that survives prolonged multi-drug treatment in mice
Source: mBio. 2023 Oct 31;14(6):e02363-23. doi: 10.1128/mbio.02363-23 (PMC10746229; doi:10.1128/mbio.02363-23)
Supplement: Supplemental Information — Additional information on methods and results. [file mbio.02363-23-s0001.docx]

**SUPPLEMENTAL INFORMATION**

**Transcriptional adaptation of *Mycobacterium tuberculosis*that survives prolonged multi-drug treatment in mice**

Elizabeth A Wynn,***** Christian Dide-Agossou,***** Matthew Reichlen, Karen Rossmassler, Reem Al Mubarak, Justin J Reid, Samuel T Tabor, Sarah E M Born, Monica R Ransom, Rebecca M Davidson, Kendra N Walton, Jeanne B Benoit, Amanda Hoppers, Dorothy E Loy, Allison A Bauman, Lisa M Massoudi, Gregory Dolganov, Michael Strong, Payam Nahid, Martin I Voskuil, Gregory T Robertson, Camille M Moore,^†^ Nicholas D Walter^†^

***** These authors contributed equally to this work (EAW, CD-A)

† These authors contributed equally to this work (NDW, CMM)

Contents

[**1.** **Supplemental Methods** 3](#_Toc143682652)

[***1.1.*** ***Table S1****. Inclusion reference genomes.* 3](#_Toc143682653)

[***1.2.*** ***Table S2****. Exclusion reference genomes* 3](#_Toc143682654)

[***1.3.*** ***Conversion to Mtb Erdman*** 4](#_Toc143682655)

[***1.4.*** ***Evaluation of amplification bias*** 4](#_Toc143682656)

[***1.5.*** ***Evaluation of repeatability of amplification*** 5](#_Toc143682657)

[***1.6.*** ***Concordance of SEARCH-TB with conventional RNA-seq*** 5](#_Toc143682658)

[***1.7.*** ***In vitro drug experiments*** 6](#_Toc143682659)

[***1.8.*** ***Murine drug experiments*** 6](#_Toc143682660)

[***1.9.*** ***RNA extraction*** 7](#_Toc143682661)

[***1.10.*** ***Library preparation*** 8](#_Toc143682662)

[***1.11.*** ***Sequencing and bioinformatic analysis*** 9](#_Toc143682663)

[***1.12.*** ***Table S3.*** *Curated gene categories used for enrichment analysis* 10](#_Toc143682664)

[**2.** **Supplemental Results** 11](#_Toc143682665)

[***2.1.*** ***Results of SEARCH-TB design*** 11](#_Toc143682666)

[***2.2.*** ***Evaluation of amplification bias*** 12](#_Toc143682667)

[***2.3.*** ***Repeatability of amplification*** 12](#_Toc143682668)

[***2.4.*** ***Concordance of SEARCH-TB with conventional RNA-seq*** 13](#_Toc143682669)

[***2.5.*** ***Sensitivity of SEARCH-TB relative to existing methods*** 14](#_Toc143682670)

[***2.6.*** **Table S3.** Expression of efflux pumps***.*** 19](#_Toc143682671)

[***2.7.*** **Table S4*.*** Expression of drug targets. 20](#_Toc143682672)

[**3.** **References** 21](#_Toc143682673)

# **Supplemental Methods**

## ***Table S1****. Inclusion reference genomes.*

Genomes from eight *M tuberculosis* Complex strains used in the design process to assure that SEARCH-TB amplifies sequence that is common across lineages.

| Species | Strain Name | Lineage | Genbank accession number |
| --- | --- | --- | --- |
| *Mycobacterium tuberculosis* | EAI5 | L1 | NC_021740 |
| *Mycobacterium tuberculosis* | CCDC5180 | L2 | NC_017522 |
| *Mycobacterium tuberculosis* | CAS/NITR204 | L3 | NC_021193 |
| *Mycobacterium tuberculosis* | CDC1551 | L4 | NC_002755 |
| *Mycobacterium tuberculosis* | H37Rv | L4 | NC_000962 |
| *Mycobacterium tuberculosis* | Erdman | L4 | AP012340.1 |
| *Mycobacterium africanum* | GM041182 | L6 | NC_015758 |
| *Mycobacterium bovis* BCG | Pasteur1173P2 | L8 | NC_008769 |

## ***Table S2****. Exclusion reference genomes*

Genomes from 12 eukaryotes and bacteria used in the design process to assure that SEARCH-TB does not amplify sequence from non-*M tuberculosis* complex species.

| Species | Strain Name |
| --- | --- |
| *Homo sapiens* |  |
| *Mus musculus* | C57BL/6J |
| *Mycobacterium avium* | H87 |
| *Mycobacterium abscessus* | ATCC19977 |
| *Mycobacterium kansasii* | ATCC12478 |
| *Escherichia coli* | K-12 |
| *Streptococcus mutans* | UA159 |
| *Rhodococcus equi* | 103S |
| *Pseudomonas aeruginosa* | PAO1 |
| *Prevotella intermedia* | 17 |
| *Neisseria bacilliformis* | AFAY01 |
| *Nocardia asteroids* | FOTX01 |

## ***Conversion to Mtb Erdman***

*Mtb* H37Rv genes targeted by the SEARCH-TB panel were mapped to the Erdman strain and genes without an adequate match were removed from further analysis. The Basic Local Alignment Search Tool (BLAST)^1^ was used to find matches between the panel primer sequences and the Erdman genome (annotated genome obtained from RefSeq; annotation date: 06/07/2020). Genes were excluded from the panel if their corresponding primers did not map to a gene in the Erdman genome, there were more than 2 mismatches between either primer and the best match to the Erdman genome, or the location of the matches in the Erdman genome for a set of primers were more than 160 base pairs apart.

## ***Evaluation of amplification bias***

We tested for amplification bias (*i.e.,* differences in amplification efficiency between primer pairs targeting different *Mtb* transcripts) by sequencing *Mtb* genomic DNA with SEARCH-TB. The value of DNA for this purpose is that each gene is present as a single copy in the genome. To avoid overloading the amplification steps with template material, 0.001 ng *Mtb* H37Rv genomic DNA was diluted in water and sequenced in duplicate using SEARCH-TB. The raw counts for each sample were transformed to counts per million (CPM). In the absence of amplification bias (*i.e.,* if all primers amplified with identical efficiency), the expected CPM would be one million divided by the number of targets. We compared observed CPM for each target with the ideal CPM expected in the absence of bias. Results are shown in Section 2.2, Fig. S3.

## ***Evaluation of repeatability of amplification***

The repeatability of amplification was evaluated by evaluating samples with a known amount of *Mtb* RNA. To prepare these samples, we spiked 0.001 ng *Mtb* RNA, collected at log-phase growth into 1 ng human lung RNA purchased from Invitrogen. Aliquots of this spiked-in mixture were sequenced with SEARCH-TB. Results are shown in Section 2.3, Fig. S4.

## ***Concordance of SEARCH-TB with conventional RNA-seq***


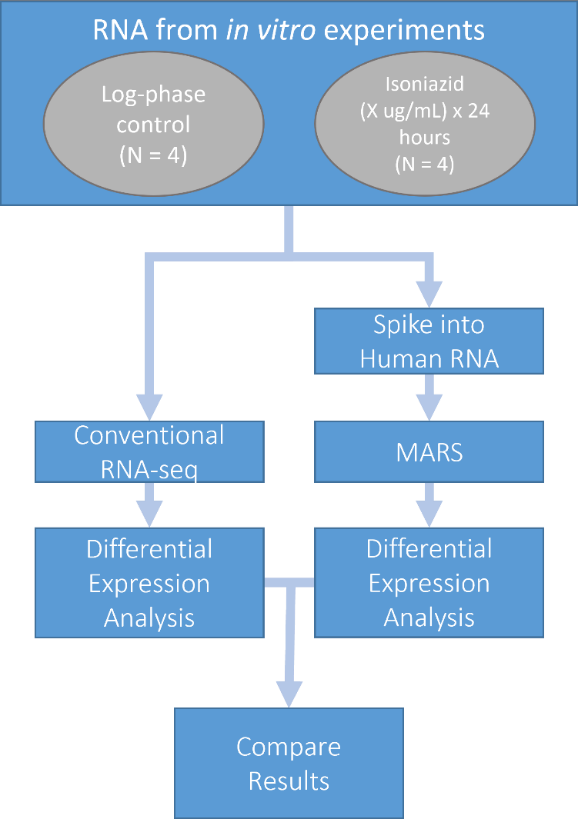
Our purpose was to determine if SEARCH-TB provided the same biological information that would be obtained by conventional RNA-seq (**Fig S1**). *Mtb* was grown to early log phase and exposed to isoniazid (0.1 ug/mL) for 24 hours. RNA was extracted from control and isoniazid-treated samples. Control and isoniazid-treated RNA aliquots (N=4 each) were prepped using the Illumina TrueSeq protocol and sequenced on the NovaSeq as described in Sections 1.10-1.11 below. We described this sequencing strategy which did not include *Mtb-*targeted amplification as our conventional RNA-seq reference. Additional control and isoniazid-treated RNA aliquots (N=4 each) were also spiked into human RNA at a ratio of 1:1,000. Libraries were prepped via SEARCH-TB and sequenced on the NovaSeq as described in Sections 1.10-1.11. For both the conventional RNA-seq and SEARCH-TB results, we calculated differential expression between the control and isoniazid-treated samples using edgeR. We evaluated the concordance of fold-changes and differentially expressed gene lists between conventional RNA-seq and SEARCH-TB.

**Fig. S1**. Conceptual diagram for comparison of SEARCH-TB with conventional RNA-seq.

## ***In vitro drug experiments***

For *in vitro* drug exposure, *Mtb* strains H37Rv and/or Erdman were exposed to 0.1 μg/ml isoniazid or HRZE with individual component concentrations of 0.1, 0.1, 3.2, and 4.0 μg/ml for isoniazid, rifampin, pyrazinamide, and ethambutol, respectively. Drug exposure experiments were carried out as described in Walter et al., 2021.^2^ Cultures were collected for RNA after 24 hour isoniazid exposure and 4 days and 8 days of HRZE exposure. Drugs were obtained from the following suppliers: isoniazid (Sigma, cat# I1377), rifampicin (Chem-Impex Int’l, cat# 00260), pyrazinamide (Sigma, cat# PHR1576), ethambutol (Sigma, PHR1930). Drug stocks were prepared in water (isoniazid, ethambutol) or DMSO (rifampicin, pyrazinamide) at 10 mg/ml, filter sterilized, and stored at -80^o^C. Drugs were diluted to 1000X working concentration immediately prior to use.

## ***Murine drug experiments***

Female BALB/c, 6- to 8-week-old mice, were infected with *Mtb* Erdman by high dose aerosol using a GlasCol chamber as described previously (PMID: 34006838). Treatment started 11 days later (D0). Mice were sacrificed for lung CFU counts on the day after infection and D0 to determine the number of CFU implanted and the number present at the start of treatment, respectively. Lungs were dissected aseptically, and flash frozen in liquid nitrogen before processing. Rifampin (R) was administered at 10 mg/kg. Isoniazid (H) was administered at 10 mg/kg. Pyrazinamide (Z) was administered at 150 mg/kg. Ethambutol (E) was administered at 100 mg/kg. All drugs were prepared in sterile water and given by oral gavage 5 of 7 days per week. HZE was administered in combination 1 hour after mice received R. Assessment of bactericidal activity were based on lung CFU counts after 2 or 4 weeks of treatment. At each time point, lungs were dissected aseptically, and the left lung, and inferior and post-caval lobes (2/3rds of the total lung by mass) were homogenized in 4.5 ml PBS + 10% [w:v] bovine serum albumin (BSA). Lung homogenates were plated in serial dilutions on 0.4% [w:v] charcoal-supplemented 7H11 agar supplemented with 10% oleic acid, BSA, sodium chloride, dextrose, and catalase (OADC) and with selective antibiotics: cycloheximide (10 mg/L), carbenicillin (50 mg/L). The remaining superior and middle lung lobes (1/3rd of the total lung by mass), were recovered and flash frozen under liquid nitrogen for RNA preservation.

## ***RNA extraction***

*In vitro* cells were collected in a 5M GTC-TCEP solution^3^ at a ratio of one volume culture to two volumes GTC-TCEP. Cultures were mixed and incubated at room temperature for five minutes. Mycobacterial cells were pelleted by centrifugation at 4000 rpm for five minutes. Cell pellets were resuspended in 1 mL RLT+ BME (Qiagen, 1% BME) with 0.5 mL 0.1 mm glass beads. Cells were lysed by beadbeating using a FastPrep-24 (MP Biomedical) at 4.0 m/s for 30 seconds four times, resting on ice for two minutes each time.

Murine tissue samples were snap frozen in 7 mL Precellys tubes (Bertin) under liquid nitrogen. To each tissue sample, 1.5 mL GTC-TCEP buffer^3^ was added and samples were homogenized at 7,200 rpm for 16s using a Precellys Evolution (Bertin). Homogenates were incubated at room temperature for 5 minutes while protected from light. Unlysed mycobacterial cells were pelleted at 10,000g for 3 minutes, then resuspended in 0.8 mL RLT+ BME buffer (Qiagen, TCEP concentration 0.75 mM). Mycobacterial cells were lysed on a Precellys Evolution (Bertin) at 6,500 rpm for 30s three times, resting on ice for 5 minutes each time, and stored at -80⁰C.

After storage at -80⁰C, *in vitro* and murine lysates were thawed, and RNA was purified using the Maxwell simplyRNA cells kit (*in vitro* samples) or simplyRNA tissue kit (murine samples) using the Maxwell RSC instrument following the manufacturer’s instructions with the following modifications. Additional kit DNase was added at twice the manufacturer’s recommended amount. Purified RNA was quantified using the Quantifluor RNA system (Promega).

## ***Library preparation***

This project prepared two library types (SEARCH-TB and Illumina RNA-seq). This section will describe these two library preparations sequentially.

Preparation of SEARCH-TB libraries

Samples were prepared for SEARCH-TB using Illumina’s AmpliSeq for Illumina Custom and Community RNA Panels kit, according to the Illumina’s recommendations except where noted. RNA was diluted to a concentration of 15-30 ng/uL, and reverse transcribed. cDNA targets were amplified with the SEARCH-TB panel with the following thermocycling conditions: 99°C for 2 minutes, 18 cycles of 99°C for 15 seconds and 60°C for 8 minutes, hold at 10°C. Amplicons were partially digested, indexes were ligated, and the library was cleaned. The library was amplified, with the following thermocycling conditions: 98°C for 2 minutes, 9 cycles of 98°C for 15 seconds and 64°C for 1 minute, hold at 10°C. The library was cleaned a second time, quantified via Qubit using the dsDNA kit (Invitrogen), and evaluated for quality via TapeStation with the High Sensitivity D1000 ScreenTape (Agilent). Libraries were diluted to the same concentration and checked for intact sequencing adaptors via qPCR. Inserts were 124-139 bp, yielding amplicons of approximately 280 bp.

RNA-seq libraries were prepared with 100 ng of high-RIN RNA using the Truseq Stranded Total RNA-seq library prep kit with RiboZero Plus as per protocol (Illumina). Briefly, samples were depleted of rRNA with the RiboZero Plus depletion kit, denatured and fragmented, followed by cDNA generation and adaptor ligation. Libraries were targeted for sequencing of 10 million paired-end 2x150 bp reads on a NovaSeq 6000 instrument (Illumina).

## ***Sequencing and bioinformatic analysis***

Libraries were sequenced on an Illumina NovaSeq6000 at the University of Colorado Anschutz Medical Campus Genomics Shared Resource, 2x150. 8,886,900 - 92,226,565 pairs of raw sequences were obtained per library. Bioinformatics analyses were performed on the Health Data Compass Eureka v1.0 at the University of Colorado Anschutz Medical Campus. Adaptors and bar codes were removed, and sequences were quality-trimmed with Skewer, with a minimum Qscore of 20 and a length between 50-175 bp.^4^ FastQC was used to confirm the size distribution and high quality of the remaining sequences. High-quality sequences were mapped to *Mtb* Erdman using Bowtie2 v2.4.0 using the default parameter.^5^ Mapped sequences were counted using HtSeq v1.0 with the default parameters.^6^

## ***Table S3.*** *Curated gene categories used for enrichment analysis*

Gene categories curated from the literature which were used in enrichment analysis. Along with the reference, the total number of genes from each source along with the number of genes from each source which were in the SEARCH-TB assay is given. Other gene sets used for enrichment analysis are available in Cole et al., 1998.^7^

| **Category** | **# of Genes in Assayed** | **# of Genes in Category** | **Source** |
| --- | --- | --- | --- |
| ABC transporters - Type I Sugar Import | 12 | 12 | (Soni, Dubey, & Bhatnagar, 2020)^8^ |
| ABC transporters Type I phosphate | 8 | 8 | (Soni, Dubey, & Bhatnagar, 2020)^8^ |
| Antitoxins | 72 | 76 | (Shao et al., 2011)^9^ |
| Arabinogalactan (AG) | 18 | 19 | (Abrahams & Besra, 2018)^10^ |
| Beta Oxidation | 18 | 18 | (Schnappinger et al., 2003)^11^ |
| Cell wall synthesis | 40 | 40 | (Kirksey et al., 2011)^12^ |
| Cholesterol A and B ring degradation | 10 | 10 | (Pawełczyk et al., 2021)^13^ |
| Cholesterol side chain degradation | 33 | 33 | (Pawełczyk et al., 2021)^13^ |
| DNA replication and repair | 25 | 27 | (Ditse, Lamers, & Warner, 2017)^14^ |
| DosR | 48 | 48 | (Voskuil et al., 2003)^15^ |
| Drug targets | 40 | 40 | (“Working Group for New TB Drugs.,” 2021)^16^  (Shetye, Franzblau, & Cho, 2020)^17^ |
| Efflux Pumps and Transports | 25 | 26 | (Remm, Earp, Dick, Dartois, & Seeger, 2022)^18^ |
| Enduring Hypoxic Response | 149 | 161 | (Rustad, Harrell, Liao, & Sherman, 2008)^19^ |
| Esterases (Lip family) | 20 | 22 | (Tallman, Levine, & Beatty, 2016)^20^ |
| Esterases (non-Lip family) | 13 | 13 | (Tallman, Levine, & Beatty, 2016)^20^ |
| ESX1 | 18 | 19 | (Gröschel, Sayes, Simeone, Majlessi, & Brosch, 2016)^21^ |
| ESX2 | 12 | 12 | (Gröschel, Sayes, Simeone, Majlessi, & Brosch, 2016)^21^ |
| ESX3 | 9 | 11 | (Gröschel, Sayes, Simeone, Majlessi, & Brosch, 2016)^21^ |
| ESX5 | 11 | 15 | (Gröschel, Sayes, Simeone, Majlessi, & Brosch, 2016)^21^ |
| Fatty Acid Synthases II | 8 | 9 | (Duan, Xiang, & Xie, 2014)^22^ |
| kstR1 regulon | 70 | 71 | (Wipperman, Sampson, & Thomas, 2014)^23^ |
| kstR2 regulon | 14 | 15 | (Wipperman, Sampson, & Thomas, 2014)^23^ |
| LAM | 14 | 15 | (Batt, Burke, Moorey, & Besra, 2020)^24^ |
| mmpL | 14 | 14 | (Domenech, Reed, & Barry, 2005)^25^ |
| Mycobactin Biogenesis | 10 | 10 | (Quadri, Sello, Keating, Weinreb, & Walsh, 1998)^26^ |
| Mycolic acid modification | 12 | 12 | (Marrakchi, Lanéelle, & Daffé, 2014)^27^ |
| NADH dehydrogenase type I | 12 | 14 | (Cook, Hards, Vilchèze, Hartman, & Berney, 2014)^28^ |
| Oxidative Stress | 48 | 49 | (Voskuil, Bartek, Visconti, & Schoolnik, 2011)^29^ |
| PDIM | 20 | 20 | (Rens, Chao, Sexton, Tocheva, & Av-Gay, 2021)^30^ |
| Peptidoglycan (PG) | 32 | 34 | (Maitra et al., 2019)^31^ |
| Sigma Factors | 12 | 13 | (Lew, Kapopoulou, Jones, & Cole, 2011)^32^ |
| Stringent Response | 123 | 147 | (Dahl et al., 2003)^33^ |
| Toxin-Antitoxin | 146 | 152 | (Shao et al., 2011)^9^ |
| Toxins | 74 | 76 | (Shao et al., 2011)^9^ |
| Transcription Factors | 187 | 198 | (Lew, Kapopoulou, Jones, & Cole, 2011)^32^ |
| Trehalose | 10 | 10 | (Wilson et al., 1999)^34^ |
| Triacylglycerol Synthases | 14 | 16 | (Thanna & Sucheck, 2016)^35^ |
| Universal stress proteins | 9 | 10 | (Lew, Kapopoulou, Jones, & Cole, 2011)^32^ |
| Zur regulon | 17 | 20 | (Dow et al., 2021)^36^ |

# **Supplemental Results**

## ***Results of SEARCH-TB design***

Primers were designed to amplify 3,733 (92.6%) of 4,031 CDS in the *Mtb* H37Rv genome. Primers for 95 genes failed to amplify in initial testing with genomic DNA and were excluded from analysis. Enrichment analysis showed that these 95 genes were not overrepresented in any biological categories used in this paper (adj-*P*=1 for all gene sets). Additionally, we excluded 70 genes that were not effective in the *Mtb* Erdman strain used in murine and *in vitro* experiments. The final panel analyzed targeted 3,568 *Mtb* genes (**Fig. S2**).

| 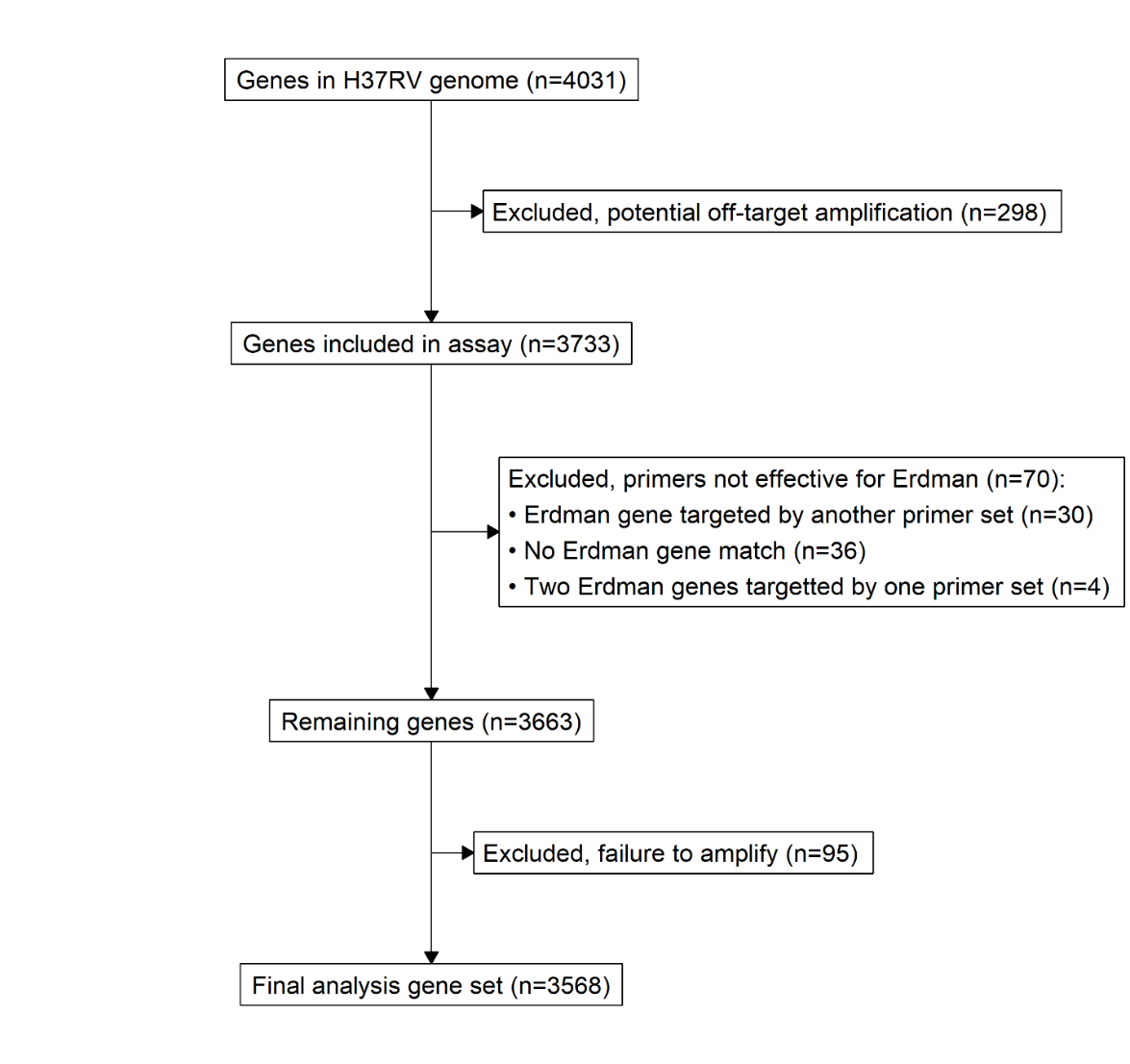 |
| --- |
| **Figure S2. Flow diagram of design/testing of SEARCH-TB.** A custom assay was created using Illumina AmpliSeq technology with the H37RV *Mtb* strain used as a reference along with eight other *Mtb* reference strains. To avoid off-target amplification of non-*Mtb­* organisms, genes with similar sequences in 12 exclusion genomes were removed. After designing the assay, primers were compared to the Erdman strain and genes which corresponded to primers not effective on the Erdman strain were removed from further analysis. Finally, genes which failed to adequately amplify in quality assurance experiments with genomic DNA were also excluded from downstream analysis. This resulted in 3,568 genes used in the final analysis. |

## ***Evaluation of amplification bias***

Using gDNA (a matrix in which each target sequence should be present in equal abundance), we quantified deviation from the ideal value that would be expected if all primers had identical amplification efficiency. Of all primer pairs, 78% were within one log_2_ fold change of the ideal value (**Fig. S3**).

| 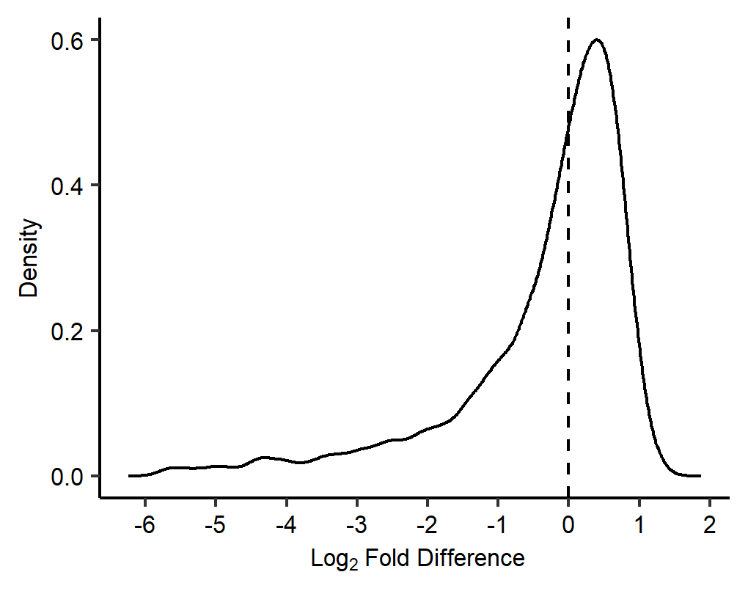 |
| --- |
| **Fig. S3. Evaluation of amplification bias.** Distribution of log_2_ fold differences of normalized gDNA SEARCH-TB expression data relative to the value expected if there were no amplification bias. Zero (dashed vertical line) represents no amplification bias. |

## ***Repeatability of amplification***

SEARCH-TB results were highly repeatable among spike-in technical replicates (**Fig. S4a**). The batch effect between replicates prepared and sequenced over time was minimal relative to the treatment effect (**Fig. S4b**).

| 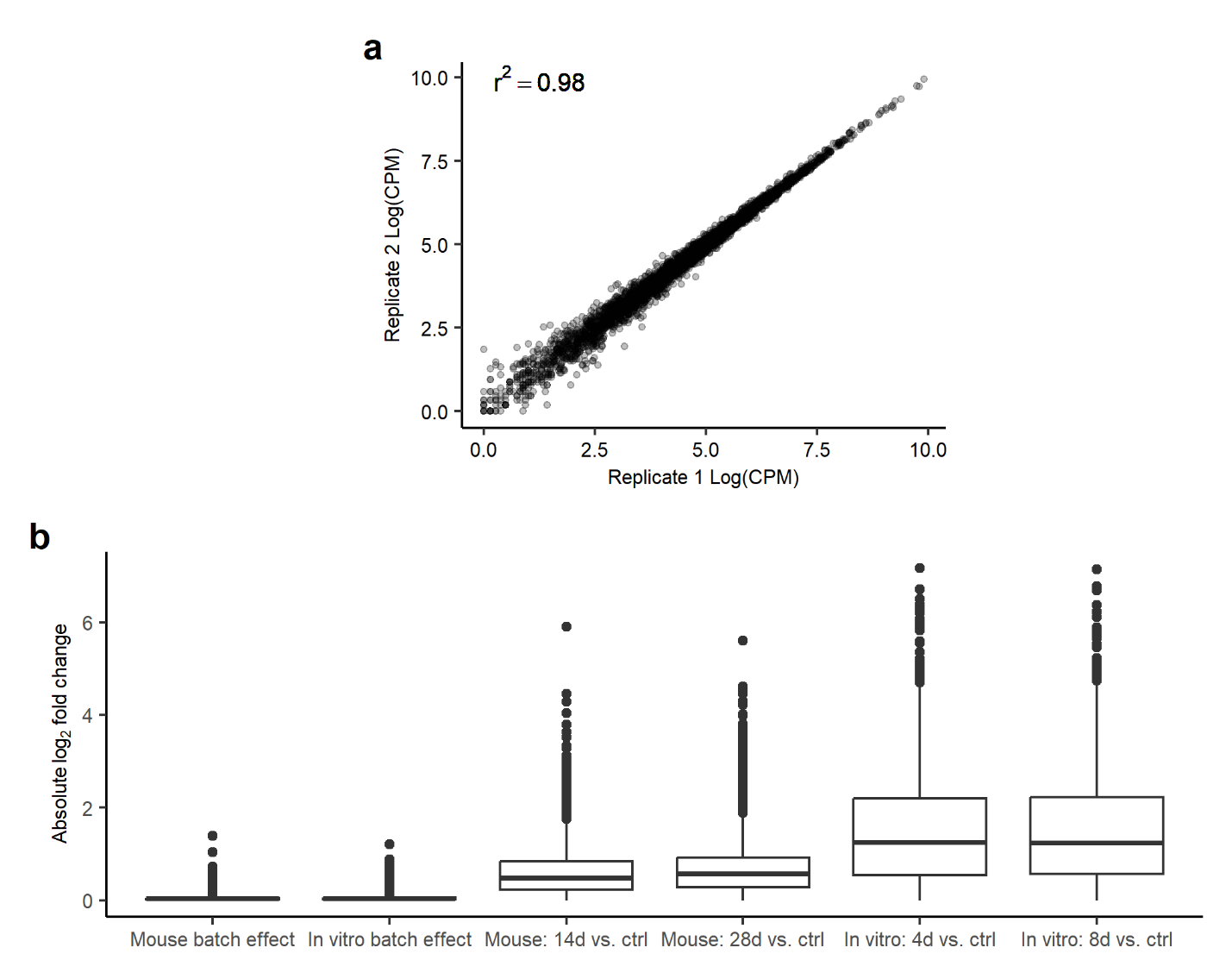 |
| --- |
| **Fig. S4. Repeatability of amplification. a.** Evaluation of repeatability of SEARCH-TB showing normalized expression data (log counts per million) for two technical replicates in which *Mtb* RNA was spiked into human lung RNA. **b.** Batch effect values were calculated by averaging the absolute log2 fold difference in gene expression values between replicate pairs across 19 pairs of replicate samples that underwent library prep and sequencing via SEARCH-TB twice. Treatment effect values were extracted from edgeR models comparing treatment groups. This demonstrates that the magnitude of the batch effect was very small relative to the treatment effect. |

## ***Concordance of SEARCH-TB with conventional RNA-seq***

RNA from *in vitro* exposure showed a similar direction and scale of differential expression when sequenced via SEARCH-TB and conventional RNA-seq. The gene sets identified as differentially expressed by the two platforms strongly overlapped. The fold-changes quantified by the two platforms showed strong agreement (*R^2^*=0.82), indicating that while SEARCH-TB is uniquely capable of profiling in drug-treated animals, both platforms provide the same biological information in *in vitro* RNA.

## ***Sensitivity of SEARCH-TB relative to existing methods***

Profiling the *Mtb* transcriptome in vivo requires an enrichment step (in which *Mtb* transcripts are captured or amplified) and a quantification step (e.g., microarray, qPCR or RNA-seq). Microarray is now a relatively archaic quantification method with lower sensitivity than qPCR or RNA-seq. qPCR typically interrogates a relatively small number of genes. A historical platform that performed individual qPCR reactions for >2,400 individual *Mtb* genes has been phased out due to cost, extreme labor requirement and variation in amplification efficiency. To narrow our scope, we will therefore restrict our comparison to only RNA-seq methods.

We identified six manuscripts that used RNA-seq to evaluate the *Mtb* transcriptome in laboratory animals or humans. A brief narrative synopsis is provided below. We present these in subjective order of relevance to SEARCH-TB with the most relevant comparator presented first.

For reference, our threshold for considering *Mtb* transcriptome data “analyzable” is at least one million reads mapping to *Mtb* CDS. We set one million *Mtb* reads as a quality control threshold because experience has shown that this generally results in at least 10 reads for 90% of our 3,568 targets). When coverage is substantially lower than one million reads, the number of reads per transcript can be quite low, adding stochastic error that affects data quality.

For each manuscript, where available, we aimed to summarize: (1) the depth with which samples were sequenced (i.e., average reads), (2) the percentage of reads mapped to *Mtb* CDS, (3) the absolute number of reads mapped to *Mtb* CDS and (4) the number of *Mtb* genes with detectable expression and (5) the percentage of *Mtb* genes with detectable expression.

These performance metrics for SEARCH-TB are illustrated below for murine samples treated with HRZE.

| **Metric** | | **Day 0** | **Day 14** | **Day 28** |
| --- | --- | --- | --- | --- |
| Average total reads | | 23,929,743 | 58,181,611 | 57,303,203 |
| % of reads mapping to *Mtb* CDS | | 84.9% | 76.1% | 55% |
| Average number of reads mapping to *Mtb* CDS | | 17,429,313 | 33,813,658 | 19,894,335 |
| Number of *Mtb* genes with detectable expression | | 3,565 | 3,565 | 3,561 |
| Percentage of all *Mtb* genes with detectable expression * | | 87.6% | 87.6% | 87.5% |
| Percentage of *Mtb* genes included in the SEARCH-TB panel with detectable expression** | | 99.9% | 99.9% | 99.8% |
| * Number of genes detected divided by number of annotated genes (4,071)  ** Number of genes detected divided by number of genes in the SEARCH-TB panel (3,568). | | | |  |

**Cornejo-Granados et al., 2021^37^**

**“Targeted RNA-Seq reveals the *M. tuberculosis* transcriptome from an *in vivo* infection model”**

The authors used a method of differential cell lysis and probe-based ribosomal depletion to profile *Mtb* in the treatment-naïve BALB/c mouse after high-dose intratracheal infection (2.5 x 105 organisms) with *Mtb* H37Rv. Mice were sacrificed on infection day 21 and lungs were collected for RNA analysis. The publication does not report lung CFU at the time of sacrifice but experience with this model would suggest a very high bacterial burden in the untreated mouse. This method detected transcripts for 702 genes (~18% of the *Mtb* genome).

**Cerezo-Cortés et al., 2022**^38^

**“Close related drug-resistance Beijing isolates of *Mycobacterium tuberculosis* reveal a different transcriptomic signature in a murine disease progression model”**

Using the same method demonstrated by Cornejo-Granados above, the authors compared transcription of two clinical *Mtb* Beijing strains (identified as BL-323 and BC-391) in the untreated BALB/c mouse. The starting bacterial inoculum and CFU burden at subsequent days was not described. Mice were sacrificed 3, 14, 28, and 60 following infection.

Table 1 of the manuscript shows that on average ~58,000 reads mapped to *Mtb* transcripts per sample. This is ~20-fold lower than our own million read per sample threshold for analyzable results. The table shows that on average 33% of *Mtb* CDS had detectable expression, implying that 66% of genes were undetected.

| **Reads/Genes** | **BL-323 Day PI** | | | **BC-391 Day PI** | | | |
| --- | --- | --- | --- | --- | --- | --- | --- |
|  | **3** | **14** | **28** | **3** | **14** | **28** | **60** |
| **Number of reads mapped to CDS *Mtb*** | 52,167 | 58,889 | 58,970 | 49,105 | 76,026 | 56,595 | 55,783 |
| **Number and % of *MTB* genes mapped** | 1,148 (30%) | 1,429 (37%) | 1,493 (39%) | 1,323 (33%) | 705 (18%) | 1,357 (34%) | 1,623 (41%) |

**Skvortsov, Ignatov, Majorov, Apt, & Azhikina, 2013^39^**

**“*Mycobacterium tuberculosis* transcriptome profiling in mice with genetically different susceptibility to tuberculosis”**

The authors performed coincidence cloning followed by RNA-seq in two mouse strains (I/St and C57BL/6YCit) 4 and 6 weeks after low-dose aerosol infection with 100-200 CFU of H37Rv. The publication does not report lung CFU at the time of sacrifice. Mice were not administered anti-microbial treatment.

A total of three libraries were prepared: I/St mice at week 6 and C57BL/6YCit at weeks 4 and 6. Table 1 of the manuscript shows that on average ~31,000 reads mapped to *Mtb* (it is unclear whether this corresponds to CDS or any *Mtb* feature). As noted above, this is many-fold lower than transcriptome data we would consider analyzable. The table shows that on average 1,435 genes were detected, representing <50% of the genome.

| **Library** | **CC4 (RES)** | **CC6 (SUS)** | **CC6 (RES)** |
| --- | --- | --- | --- |
| *Mtb*-specific reads (unique) | 14,990 | 43,618 | 34,234 |
| Genes expressed (number of reads > 0) | 1,012 | 1,353 | 1,940 |

**Pisu, Huang, Grenier, & Russell, 2020^40^**

**“Dual RNA-Seq of *Mtb*-Infected macrophages *in vivo* reveals ontologically distinct host-pathogen interactions”**

The authors isolated alveolar and interstitial macrophages from C57BL/6J WT mice infected with *Mtb* Erdman smyc’::mCherry as follows. Lung tissue was dissociated and filtered, red blood cells were lysed, resultant suspensions were incubated with fluorophore-conjugated antibodies to label macrophages, samples were washed and sorted via flow cytometry and RNA was preserved in Trizol. RNA was extracted, RNA-seq libraries were constructed and sequenced.

Table 1 of the manuscript shows that RNA alveolar macrophages were sequenced deeply (average 142 M reads/sample). On average, ~1.1 M reads mapped to *Mtb*, meeting our threshold for analyzable transcriptional data. This resulted in detection of 97% and 96% of *Mtb* genes in alveolar and interstitial macrophages, respectively.

| **Sample** | **Raw Reads** | **Aligned to *Mtb* Erdman** | **% of Raw Reads** |
| --- | --- | --- | --- |
| AM 1 ~25,000 infected cells | 131.5M | 1.2M | 0.90% |
| AM 2 ~18,000 infected cells | 176.5M | 1.3M | 0.75% |
| AM 3 ~12,000 infected cells | 309.8M | 0.95M | 0.30% |
| IM 1 ~25,000 infected cells | 140.6M | 1.2M | 0.85% |
| IM 2 ~40,000 infected cells | 47.5M | 0.9M | 1.90% |
| IM 3 ~35,000 infected cells | 83.7M | 1M | 1.20% |

A key difference between the Pisu method and SEARCH-TB is that the Pisu method quantifies *Mtb* expression in isolated macrophage subtypes whereas SEARCH-TB evaluates *Mtb* expression in whole lung. The Pisu method is an extraordinary technical accomplishment designed and applied for analysis of host-pathogen interaction. The extensive sample manipulation may not be suited to routine use for drug evaluation. We are unaware of application of the Pisu method to drug-treated animals. By contrast, SEARCH-TB was designed to be a pharmacodynamic marker used to evaluate drug effects and has demonstrated capability to elicit bacterial phenotypes after prolonged treatment.

**Lai et al., 2021**^41^

**“Transcriptomic characterization of tuberculous sputum reveals a host Warburg Effect and microbial cholesterol catabolism”**

The authors extracted RNA from the sputum of 17 patients with untreated active pulmonary TB. Libraries were constructed without pathogen-specific selection or enrichment and sequenced deeply (average 170 million reads/sample).

The number of reads mapping to *Mtb* was low, ranging from 10^3^ to 10^5^. The maximal number is 100-times lower than our threshold for analyzable transcriptional data. The authors state “seven sample had sufficient read coverage (>4 x 10^4^) to quantify transcript abundance for >50% of the M. tuberculosis genome. The low coverage achieved in untreated humans illustrates the sensitivity challenge that SEARCH-TB was designed to address.

**Shaikh et al., 2021**^42^

**“Early phase of effective treatment induces distinct transcriptional changes in Mycobacterium tuberculosis expelled by pulmonary tuberculosis patients”**

The authors evaluated *Mtb* expression in aerosol generated by patients with active pulmonary TB (using capture membranes in N95 masks). Samples were collected prior to treatment initiation and with up to 14 days of HRZE. RNA was extracted and libraries were prepared without pathogen-specific selection or enrichment. Fifty patients were enrolled but results were presented for only 19.

Table S2 of the manuscript shows that the percentage of reads that mapped to *Mtb* was relatively low (on average 1.2%). The average number of *Mtb* reads was ~50% of our threshold for analyzable results. It is unclear if this represents *Mtb* CDS or reads mapped to any *Mtb* feature. The number of transcripts detected ranged from ~50% of the genome in pre-treatment samples to ~10% on day 14.

|  | Treatment Time points | | | | | |
| --- | --- | --- | --- | --- | --- | --- |
|  | **Pre-Rx** | **Rx-1D** | **Rx-3D** | **Rx-5D** | **Rx-7D** | **Rx-14D** |
| *Mtb* percentage | 1.51% | 2.7% | 0.66% | 1.44% | 0.55% | 0.44% |
| *Mtb* Specific reads | 590,217 | 935,018 | 240,696 | 541,793 | 174,478 | 195,536 |
| Genes covered | 1988 | 833 | 303 | 273 | 425 | 410 |

In summary, the six manuscripts above highlight the challenge of obtaining *Mtb* transcriptome data in a eukaryotic background. Despite very high sequencing depth used in most studies, previous methods have resulted in a low percentage of reads mapping to *Mtb* CDS and a low percentage of *Mtb* genes that have detectable expression. For most methods, the number of reads mapped to *Mtb* CDS is well below the threshold we consider analyzable.

**No existing reports of RNA-seq of *Mtb* gene expression in drug-treated animals**

We were unable to identify previous studies that used RNA-seq or any other transcriptional profiling method to characterize *Mtb* phenotypes in drug-treated mice or other laboratory animals.

The challenge of profiling the bacterial transcriptome increases as drug treatment decreases *Mtb* burden. Additionally, our unpublished data indicate that the abundance of mRNA per bacillus declines after drug exposure. For example, at aerobic log-phase growth, ~8% of all RNA-seq reads are messenger RNA. With rifampin exposure, the percentage of reads that map to mRNA decreases to 3%, 1.1% and 0.7% after 4 hours, 2 days and 4 days, respectively Thus, as the bacterium transitions to a drug-stressed quiescent phenotype with less transitional activity, the challenge of transcriptional profiling likely increases.

We believe that the combination of decreasing bacterial burden plus transition to a “harder-to-profile” phenotype is likely the reason that the current manuscript is apparently the first to profile *Mtb* in laboratory animals after prolonged treatment. Given the limited sensitivity of existing methods in untreated mice described above, these methods are likely not capable of quantifying *Mtb* expression in untreated mice with much lower burdens. SEARCH-TB represents a substantial improvement in sensitivity that makes quantification of the *Mtb* transcriptome in drug-treated animals possible.

## **Table S3.** Expression of efflux pumps***.***

Fold-change and adjusted *P-*values for efflux pumps with potential drug-transporting capacity as identified in Remm, *et.al.*^18^

| **Transporter Superfamily** | **Pump/Gene RV (symbol)** | **Mouse day 28** | | **In vitro day 8** | |
| --- | --- | --- | --- | --- | --- |
|  |  | **log_2_ FC** | **adj-*P* val** | **log_2_ FC** | **adj-*P* val** |
| RND transporter | MmpL5-MmpS5 |  |  |  |  |
|  | Rv0676c (mmpL5) | -0.34 | **2.79E-02** | -0.12 | 5.59E-01 |
|  | Rv0677c (mmpS5) | -0.67 | **2.59E-03** | 0.66 | **1.79E-02** |
| MFS transporter | Rv1258c (Tap) |  |  |  |  |
|  | Rv1258c | 1.04 | **3.05E-08** | 1.62 | **1.05E-11** |
| SMR transporters | Rv3065 (Mmr) |  |  |  |  |
|  | Rv3065 (mmr) | 0.43 | **4.66E-02** | 0.19 | 4.94E-01 |
| ABC transporter | Rv2686c-2688c |  |  |  |  |
|  | Rv2686c | 1.20 | **4.07E-08** | 2.70 | **4.21E-21** |
|  | Rv2687c | 1.05 | **8.24E-08** | 2.52 | **6.90E-23** |
|  | Rv2688c | 1.22 | **1.27E-11** | 3.31 | **7.67E-41** |
| ABC transporter | Rv0194 |  |  |  |  |
|  | Rv0194 | 0.70 | **4.06E-06** | 2.37 | **9.58E-33** |
| ABC transporter | Rv2936-2938 (DrrABC) |  |  |  |  |
|  | Rv2936 (drrA) | -1.82 | **4.44E-18** | -1.41 | **9.77E-08** |
|  | Rv2937 (drrB) | -1.00 | **1.09E-08** | -0.64 | **4.12E-03** |
|  | Rv2938 (drrC) | -0.45 | **4.41E-03** | 0.70 | **3.55E-04** |
| MFS transporter | Rv1634 |  |  |  |  |
|  | Rv1634 | 0.40 | **1.40E-02** | 1.29 | **2.31E-10** |
| MFS transporter | Rv2333c (Stp) |  |  |  |  |
|  | Rv2333c (stp) |  |  |  |  |
| MFS transporter | Rv0849 |  |  |  |  |
|  | Rv0849 | 0.34 | 9.34E-02 | 1.66 | **5.46E-11** |
| MFS transporter | Rv2846c (EfpA) |  |  |  |  |
|  | Rv2846c (efpA) | -0.66 | **1.29E-04** | -1.12 | **1.49E-07** |
| ABC transporter | Rv1217c-Rv1218c |  |  |  |  |
|  | Rv1217c | 0.78 | **4.75E-05** | 0.94 | **9.75E-05** |
|  | Rv1218c | 0.89 | **2.18E-07** | 0.37 | 9.49E-02 |
| ABC transporter | Rv1456c-Rv1457c-Rv1458c |  |  |  |  |
|  | Rv1456c | 0.09 | 7.61E-01 | -0.59 | 8.48E-02 |
|  | Rv1457c | 0.24 | 2.75E-01 | -0.31 | 2.59E-01 |
|  | Rv1458c | -0.20 | 1.92E-01 | -0.25 | 2.07E-01 |
| PDIM synthesis | Rv2942 (MmpL7) |  |  |  |  |
|  | Rv2942 (mmpL7) | -0.23 | 1.29E-01 | -0.56 | **2.53E-03** |
| MFS transporter | Rv1410c (P55) |  |  |  |  |
|  | Rv1410c | -2.03 | **8.41E-21** | -2.83 | **1.48E-23** |
| ABC transporter | Rv1819c (BacA) |  |  |  |  |
|  | Rv1819c (bacA) | 0.88 | **2.07E-06** | -0.45 | 5.63E-02 |
| ABC-F subfamily | Rv2477c |  |  |  |  |
|  | Rv2477c | -1.90 | **4.23E-16** | -2.36 | **3.59E-15** |
| ABC transporter | Rv0342 and Rv0933 |  |  |  |  |
|  | Rv0342 (iniA) | 0.46 | **1.48E-03** | 1.95 | **8.68E-27** |
|  | Rv0933 (pstB) | -0.34 | 5.14E-02 | -1.44 | **1.82E-11** |

## **Table S4*.*** Expression of drug targets.

Fold-change and adjusted *P-*values for genes for targets identified by the Working Group on New TB Drugs^16^ and Shetye, *et., al.*^17^

| **Drug or class** | **Gene** | **Symbol** | **log_2_ FC** | **adj-*P-*val** | **log_2_ FC** | **adj-*P-*val** |
| --- | --- | --- | --- | --- | --- | --- |
| **Approved drugs** | | | | | | |
| **Isoniazid** | Rv1908c | katG | -1.9 | **1.6E-07** | -2.8 | **1.3E-09** |
|  | Rv1484 | inhA | -1.1 | **5.9E-09** | -2.3 | **1.1E-19** |
| **Rifamycins** | Rv0667 | rpoB | -0.1 | 7.0E-01 | -1.7 | **2.6E-05** |
| **Pyrazinamide** | Rv2043c | pncA | -0.8 | **1.4E-05** | -0.7 | **3.8E-03** |
| **Ethambutol** | Rv3795 | embB | 0.3 | 9.9E-02 | -0.6 | **8.6E-03** |
| **Fluoroquinolones** | Rv0005 | gyrB | -0.9 | **1.8E-05** | -1.4 | **3.4E-08** |
|  | Rv0006 | gyrA | -1.1 | **2.9E-09** | -2.2 | **7.5E-21** |
| **Diarylquinolones** | Rv1305 | atpE | -3.3 | **7.5E-37** | -4.6 | **1.6E-38** |
| **D-cycloserine** | Rv2981c | ddlA | -0.1 | 4.3E-01 | -0.3 | 1.1E-01 |
| **Ethionamide** | Rv3854c | ethA | -0.3 | 1.1E-01 | 0.8 | **2.5E-05** |
| **Beta-lactams (D, D-transpeptidases)** | Rv2911 | dacB2 | 0.3 | 2.5E-01 | -1.7 | **1.1E-07** |
| **Carbapenems (L, D-transpeptidases)** | Rv0116c | ldtA | -1.0 | **1.4E-07** | -0.1 | 8.4E-01 |
|  | Rv2518c | ldtB | -1.7 | **6.6E-11** | -1.7 | **6.3E-07** |
| **Clofazamine** | Rv1854c | ndh | -1.9 | **2.1E-11** | -1.6 | **3.7E-06** |
| **Investigational** | | | | | | |
| **Peptidoglycan Layer** |  |  |  |  |  |  |
| **Mur ligase inhibitors** | Rv0482 | murB | 0.7 | **1.4E-04** | -0.1 | 6.5E-01 |
|  | Rv1315 | murA | -0.3 | 9.2E-02 | -1.2 | **2.1E-08** |
|  | Rv2152c | murC | 0.3 | **3.8E-02** | 0.3 | 8.3E-02 |
|  | Rv2155c | murD | 0.2 | 4.3E-01 | 0.3 | 3.4E-01 |
|  | Rv2157c | murF | 0.2 | 3.2E-01 | -0.2 | 3.6E-01 |
| **D-alanine:D-alanine Ligase** | Rv2981c | ddlA | -0.1 | 4.3E-01 | -0.3 | 1.1E-01 |
| **Translocase 1 inhibitor** | Rv2156c | murX | 0.2 | 2.2E-01 | -0.6 | **9.4E-03** |
| **MurX inhibitors** | Rv2156c | murX | 0.2 | 2.2E-01 | -0.6 | **9.4E-03** |
| **GlcN-1-p analogues** | Rv1018c | glmU | -0.3 | 4.5E-01 | -0.1 | 7.6E-01 |
| **Polyketide synthase Pks13** | Rv3800c | pks13 | -1.8 | **1.9E-13** | -1.5 | **3.6E-07** |
| **Arabinogalactan Layer** |  |  |  |  |  |  |
| **DprE1 inhibitors** | Rv3790 | dprE1 | 0.1 | 6.9E-01 | 0.2 | 5.2E-01 |
| **Arabinosyltransferase C** | Rv3793 | embC | 0.0 | 8.8E-01 | -0.7 | **5.3E-05** |
| **WecA inhibitor** | Rv1302 | rfe | 0.5 | **2.6E-04** | 1.2 | **2.0E-11** |
| **Mycolic Acid Layer** |  |  |  |  |  |  |
| **MmpL3 inhibitors** | Rv0206c | mmpL3 | -1.1 | **2.4E-08** | -2.4 | **9.3E-21** |
| **KasA inhibitor** | Rv2245 | kasA | -2.7 | **1.7E-18** | -3.7 | **9.7E-20** |
| **Translation** |  |  |  |  |  |  |
| **Leucyl-tRNA synthase inhibitor** | Rv0041 | leuS | 0.1 | 6.7E-01 | -0.4 | **1.9E-02** |
| **DNA replication** |  |  |  |  |  |  |
| **DNA gyrase A inhibitor** | Rv0006 | gyrA | -1.1 | **2.9E-09** | -2.2 | **7.5E-21** |
| **DNA gyrase B inhibitor** | Rv0005 | gyrB | -0.9 | **1.8E-05** | -1.4 | **3.4E-08** |
| **Energy metabolism** |  |  |  |  |  |  |
| **Qcrb inhibitor (Cytochrome bc1-aa3)** | Rv2196 | qcrB | -2.2 | **4.6E-19** | -3.3 | **1.4E-24** |
| **Proteolysis & Proteostasis** |  |  |  |  |  |  |
| **clpC1 inhibitor** | Rv3596c | clpC1 | -1.5 | **2.1E-09** | -3.4 | **2.2E-23** |
| **clpP1 and clpP2 inhibitor** | Rv2460c | clpP2 | -1.7 | **2.2E-13** | -3.6 | **3.2E-31** |
|  | Rv2461c | clpP1 | -1.8 | **1.4E-12** | -3.0 | **2.7E-19** |
| **Cellular metabolism** |  |  |  |  |  |  |
| **Tryptophan synthase inhibitor** | Rv1612 | trpB | -0.3 | 5.7E-02 | -1.8 | **2.9E-16** |
|  | Rv1613 | trpA | -1.1 | **1.8E-09** | -2.0 | **1.3E-17** |
| **Aspartate decarboxylase (PZA)** | Rv3601c | panD | -1.1 | **1.8E-05** | -0.9 | **4.0E-03** |
| **Efflux transport of antibiotics** | Rv2846c | efpA | -0.7 | **1.3E-04** | -1.1 | **1.5E-07** |

# **References**

1. Altschul, S. F., Gish, W., Miller, W., Myers, E. W. & Lipman, D. J. Basic local alignment search tool. *J. Mol. Biol.* **215**, 403–410 (1990).

2. Walter, N. D. *et al.* *Mycobacterium tuberculosis* precursor rRNA as a measure of treatment-shortening activity of drugs and regimens. *Nat. Commun.* **12**, 1–11 (2021).

3. Musisi, E. *et al.* Reproducibility of the Ribosomal RNA Synthesis Ratio in Sputum and Association with Markers of Mycobacterium tuberculosis Burden. *Microbiol. Spectr.* **9**, (2021).

4. Jiang, H., Lei, R., Ding, S. W. & Zhu, S. Skewer: A fast and accurate adapter trimmer for next-generation sequencing paired-end reads. *BMC Bioinformatics* **15**, 1–12 (2014).

5. Langmead, B. & Salzberg, S. L. Fast gapped-read alignment with Bowtie 2. *Nat. Methods* **9**, 357–359 (2012).

6. Andrews, S. FastQC: A Quality Control Tool for High Throughput Sequence Data. (2010).

7. Cole, S. T. *et al.* Erratum: Deciphering the biology of Mycobacterium tuberculosis from the complete genome sequence. *Nature* **396**, 190 (1998).

8. Soni, D. K., Dubey, S. K. & Bhatnagar, R. ATP-binding cassette (ABC) import systems of *Mycobacterium tuberculosis*: target for drug and vaccine development. *Emerging Microbes and Infections* **9**, 207–220 (2020).

9. Shao, Y. *et al.* TADB: A web-based resource for Type 2 toxin-antitoxin loci in bacteria and archaea. *Nucleic Acids Res.* **39**, D606–D611 (2011).

10. Abrahams, K. A. & Besra, G. S. Mycobacterial cell wall biosynthesis: A multifaceted antibiotic target. *Parasitology* **145**, 116–133 (2018).

11. Schnappinger, D. *et al.* Transcriptional adaptation of *Mycobacterium tuberculosis* within macrophages: Insights into the phagosomal environment. *J. Exp. Med.* **198**, 693–704 (2003).

12. Kirksey, M. A. *et al.* Spontaneous phthiocerol dimycocerosate-deficient variants of *Mycobacterium tuberculosis* are susceptible to gamma interferon-mediated immunity. *Infect. Immun.* **79**, 2829–2838 (2011).

13. Pawełczyk, J. *et al.* Cholesterol-dependent transcriptome remodeling reveals new insight into the contribution of cholesterol to *Mycobacterium tuberculosis* pathogenesis. *Sci. Rep.* **11**, 1–16 (2021).

14. Ditse, Z., Lamers, M. H. & Warner, D. F. DNA replication in *Mycobacterium tuberculosis*. *Microbiol. Spectr.* **5**, (2017).

15. Voskuil, M. I. *et al.* Inhibition of respiration by nitric oxide induces a *Mycobacterium tuberculosis* dormancy program. *J. Exp. Med.* **198**, 705–713 (2003).

16. Working Group for New TB Drugs. (2021). Available at: https://www.newtbdrugs.org/pipeline/drug-targets. (Accessed: 23rd February 2023)

17. Shetye, G. S., Franzblau, S. G. & Cho, S. New tuberculosis drug targets, their inhibitors, and potential therapeutic impact. *Translational Research* **220**, 68–97 (2020).

18. Remm, S., Earp, J. C., Dick, T., Dartois, V. & Seeger, M. A. Critical discussion on drug efflux in *Mycobacterium tuberculosis*. *FEMS Microbiology Reviews* **46**, 1–15 (2022).

19. Rustad, T. R., Harrell, M. I., Liao, R. & Sherman, D. R. The enduring hypoxic response of *Mycobacterium tuberculosis*. *PLoS One* **3**, e1502 (2008).

20. Tallman, K. R., Levine, S. R. & Beatty, K. E. Small-molecule probes reveal esterases with Persistent Activity in dormant and reactivating *Mycobacterium tuberculosis*. *ACS Infect. Dis.* **2**, 936–944 (2016).

21. Gröschel, M. I., Sayes, F., Simeone, R., Majlessi, L. & Brosch, R. ESX secretion systems: Mycobacterial evolution to counter host immunity. *Nature Reviews Microbiology* **14**, 677–691 (2016).

22. Duan, X., Xiang, X. & Xie, J. Crucial components of mycobacterium type II fatty acid biosynthesis (Fas-II) and their inhibitors. *FEMS Microbiol. Lett.* **360**, 87–99 (2014).

23. Wipperman, M. F., Sampson, N. S. & Thomas, S. T. Pathogen roid rage: Cholesterol utilization by *Mycobacterium tuberculosis*. *Critical Reviews in Biochemistry and Molecular Biology* **49**, 269–293 (2014).

24. Batt, S. M., Burke, C. E., Moorey, A. R. & Besra, G. S. Antibiotics and resistance: The two-sided coin of the mycobacterial cell wall. *Cell Surface* **6**, 100044 (2020).

25. Domenech, P., Reed, M. B. & Barry, C. E. Contribution of the *Mycobacterium tuberculosis* MmpL protein family to virulence and drug resistance. *Infect. Immun.* **73**, 3492–3501 (2005).

26. Quadri, L. E., Sello, J., Keating, T. A., Weinreb, P. H. & Walsh, C. T. Identification of a *Mycobacterium tuberculosis* gene cluster encoding the biosynthetic enzymes for assembly of the virulence-conferring siderophore mycobactin. *Chem. Biol.* **5**, 631–645 (1998).

27. Marrakchi, H., Lanéelle, M. A. & Daffé, M. Mycolic acids: Structures, biosynthesis, and beyond. *Chemistry and Biology* **21**, 67–85 (2014).

28. Cook, G. M., Hards, K., Vilchèze, C., Hartman, T. & Berney, M. Energetics of respiration and oxidative phosphorylation in mycobacteria. *Microbiol. Spectr.* **2**, (2014).

29. Voskuil, M. I., Bartek, I. L., Visconti, K. & Schoolnik, G. K. The response of *Mycobacterium tuberculosis* to reactive oxygen and nitrogen species. *Front. Microbiol.* **2**, 105 (2011).

30. Rens, C., Chao, J. D., Sexton, D. L., Tocheva, E. I. & Av-Gay, Y. Roles for phthiocerol dimycocerosate lipids in *Mycobacterium tuberculosis* pathogenesis. *Microbiology (United Kingdom)* **167**, 001042 (2021).

31. Maitra, A. *et al.* Cell wall peptidoglycan in *Mycobacterium tuberculosis*: An Achilles’ heel for the TB-causing pathogen. *FEMS Microbiology Reviews* **43**, 548–575 (2019).

32. Lew, J. M., Kapopoulou, A., Jones, L. M. & Cole, S. T. TubercuList - 10 years after. *Tuberculosis* **91**, 1–7 (2011).

33. Dahl, J. L. *et al.* The role of RelMtb-mediated adaptation to stationary phase in long-term persistence of *Mycobacterium tuberculosis* in mice. *Proc. Natl. Acad. Sci. U. S. A.* **100**, 10026–10031 (2003).

34. Wilson, M. *et al.* Exploring drug-induced alterations in gene expression in *Mycobacterium tuberculosis* by microarray hybridization. *Proc. Natl. Acad. Sci. U. S. A.* **96**, 12833–12838 (1999).

35. Thanna, S. & Sucheck, S. J. Targeting the trehalose utilization pathways of *Mycobacterium tuberculosis*. *MedChemComm* **7**, 69–85 (2016).

36. Dow, A. *et al.* Zinc limitation triggers anticipatory adaptations in *Mycobacterium tuberculosis*. *PLoS Pathog.* **17**, e1009570 (2021).

37. Cornejo-Granados, F. *et al.* Targeted RNA-seq reveals the *M. tuberculosis transcriptome* from an in vivo infection model. *Biology (Basel).* **10**, 848 (2021).

38. Cerezo-Cortés, M. I. *et al.* Close related drug-resistance Beijing isolates of *Mycobacterium tuberculosis* reveal a different transcriptomic signature in a murine disease progression model. *Int. J. Mol. Sci.* **23**, 5157 (2022).

39. Skvortsov, T. A., Ignatov, D. V., Majorov, K. B., Apt, A. S. & Azhikina, T. L. *Mycobacterium tuberculosis* transcriptome profiling in mice with genetically different susceptibility to tuberculosis. *Acta Naturae* **5**, 62–69 (2013).

40. Pisu, D., Huang, L., Grenier, J. K. & Russell, D. G. Dual RNA-seq of *Mtb*-infected macrophages in vivo reveals ontologically distinct host-pathogen interactions. *Cell Rep.* **30**, 335-350.e4 (2020).

41. Lai, R. P. J. *et al.* Transcriptomic characterization of tuberculous sputum reveals a host Warburg effect and microbial cholesterol catabolism. *MBio* **12**, (2021).

42. Shaikh, A. *et al.* Early phase of effective treatment induces distinct transcriptional changes in *Mycobacterium tuberculosis* expelled by pulmonary tuberculosis patients. *Sci. Rep.* **11**, 1–13 (2021).
